# Ultrafine particulate matter exposure induces gut microbiota dysbiosis together with ER stress in the liver and worsened atherosclerosis

**DOI:** 10.1016/j.envint.2025.109964

**Published:** 2025-11-24

**Authors:** Rajat Gupta, Candace Chang, David H. Gonzalez, Priyansha Srivastava, Collin Le, Daniel P. Stefanko, Jocelyn A. Castellanos, Mohamad Navab, Srinivasa T. Reddy, Gregory A. Fishbein, Constantinos Sioutas, Jonathan P. Jacobs, Tzung Hsiai, Jesus A. Araujo

**Affiliations:** a Division of Cardiology, David Geffen School of Medicine, University of California Los Angeles, Los Angeles, CA, USA; b Department of Environmental Health Sciences, Fielding School of Public Health, University of California Los Angeles, Los Angeles, CA, USA; c Environmental and Molecular Toxicology Interdepartmental Program, University of California Los Angeles, Los Angeles, CA, USA; d Vatche and Tamar Manoukian Division of Digestive Diseases, David Geffen School of Medicine, University of California Los Angeles, Los Angeles, CA, USA; e Department of Pathology, David Geffen School of Medicine, University of California Los Angeles, Los Angeles, CA, USA; f Molecular & Medical Pharmacology, University of California Los Angeles, Los Angeles, CA, USA; g Molecular Biology Institute, University of California Los Angeles, Los Angeles, CA, USA; h University of Southern California, Viterbi School of Engineering, Los Angeles, CA, USA; i Goodman-Luskin Microbiome Center, University of California Los Angeles, Los Angeles, CA, USA; j Division of Gastroenterology, Hepatology and Parenteral Nutrition, Veterans Administration Greater Los Angeles Healthcare System, Los Angeles, CA, USA; k Henry Samueli School of Engineering, University of California Los Angeles, Los Angeles, CA, USA

**Keywords:** Atherosclerosis, Ultrafine particulate matter, Lipid peroxidation, Gut microbiota, Short chain fatty acids, ER stress, Inflammation

## Abstract

Air pollution exposure is associated with increased cardiovascular morbidity and mortality worldwide. Previous studies provide a causal relationship between exposure to particulate matter (PM) and atherosclerosis development. We have previously demonstrated increased aortic atherosclerosis and adverse metabolic effects in hyperlipidemic mice exposed to ambient ultrafine PM. However, the underlying mechanisms by which ambient PM promotes systemic effects leading to worsened atherosclerosis remain unknown. We have recently shown that the gut microbiota composition was altered in mice exposed to re-aerosolized PM in the ultrafine-size range for 10 weeks. We hypothesized that sub-chronic exposure to ultrafine PM induces gut dysbiosis in association with systemic prooxidative effects and atherosclerotic lesion development. Male apolipoprotein E knockout (ApoE^−/−^) mice were fed a chow diet and exposed to re-aerosolized PM, highly enriched in particles in the ultrafine-size range (ultrafine PM) *vs*. filtered air (FA) by inhalation (6 h/day, 3 days/week for 10 weeks). Ultrafine PM-exposed mice exhibited marked differences in the gut microbiota composition, which significantly associated with worsened atherosclerotic lesions in the innominate artery and aorta. Ultrafine PM-exposed mice also displayed significantly elevated levels of short chain fatty acids (SCFAs) in the feces, malondialdehyde (MDA) content in the liver and upregulation of hepatic antioxidant and endoplasmic reticulum (ER) stress response genes, all of which correlated with changes in the gut microbiota composition. In conclusion, inhaled PM in the ultrafine-size range induced changes in the gut microbiota composition and its metabolites, which correlated with systemic prooxidative effects, hepatic ER stress and worsened atherosclerosis.

## 1. Introduction

It is well known that ambient air pollution exposure related deaths are largely due to cardiovascular (CV) diseases (Brook et al., 2010; Rajagopalan & Landrigan, 2021), and the particulate matter (PM) components of air pollution have been most strongly correlated with adverse CV outcomes (Araujo & Nel, 2009; Bhatnagar, 2006). Epidemiological studies have demonstrated an association between exposure to PM_2.5_ (PM < 2.5 μm) with measures of atherosclerosis (Bauer et al., 2010; Diez Roux et al., 2008; Kaufman et al., 2016; Kunzli et al., 2005). Several animal studies have also reported increased atherosclerosis with 3 to 6-month intermittent (L. C. Chen & Nadziejko, 2005; Sun et al., 2005; Sun et al., 2008) or 2-month continuous exposure to PM_2.5_ in apolipoprotein E knockout (ApoE^−/−^) mice (Chen et al., 2013; Wan et al., 2014). Ultrafine particles (UFP), having almost negligible mass, represents the majority of the total number of particles within the ambient PM (Kwon et al., 2020), and are likely to concentrate the potential for adverse CV effects. In comparison to PM_2.5_, UFP are highly bioactive based on their greater particle number, larger surface-to-mass ratio, larger content of redox active compounds such as polycyclic aromatic hydrocarbons (PAHs), and greater bioavailability of chemically active constituents (Araujo & Nel, 2009). This was also demonstrated in our previous study where chow-fed ApoE^−/−^ mice exposed to concentrated UFP led to significantly increased atherosclerosis in the aorta, in a larger degree than mice exposed to PM_2.5_ and in the absence of obvious pulmonary inflammation (Araujo et al., 2008). Indeed, dissecting the underlying pathogenic mechanisms of how inhalation exposure to PM promotes atherosclerosis has been elusive.

Several studies have linked the intestinal microbiome to a wide range of human diseases, including CV, metabolic, neurological diseases and cancer (Org et al., 2015; Wu, 2016). Emerging evidence suggests that exposure to environmental pollutants lead to human gut microbiota dysbiosis (Filardo et al., 2022; Gupta et al., 2022), which may further promote systemic metabolic disorders including obesity (John & Mullin, 2016), diabetes (Han et al., 2018) and CV diseases (Stock, 2013). The gut microbiota appears to play an important role in regulating host lipid metabolism and atherosclerosis through its various metabolites including short chain fatty acids (SCFAs), bile acids, and lipopolysaccharide (LPS) (Jonsson & Backhed, 2017). It is possible that inhaled particles and/or their components could alter the gut microbiota by gaining access to the gastrointestinal tract as a result of rapid bronchial mucociliary cleansing to the oropharynx, followed by swallowing (Kreyling et al., 1999). Thus, inhaled PM has been reported to induce changes in the gut microbiome and levels of SCFAs in the colon contents (Li et al., 2020), which are gut-derived metabolites primarily consisting of acetate, propionate and butyrate, that have been shown to exert beneficial effects by counteracting dyslipidemia (Haghikia et al., 2022), hypertension (Natarajan et al., 2016), and diabetes (Zhao et al., 2018). Li et al showed that low density lipoprotein receptor knockout (Ldlr^−/−^) mice fed a high fat diet and exposed to re-aerosolized UFP by orogastric gavage for 10 weeks exhibited changes in cecal microbiome composition in association with oxidized phospholipids (Li et al., 2017). We have recently demonstrated that exposure to re-aerosolized PM in the ultrafine size range for 10 weeks led to significant alterations in microbiome profiles in the feces and small intestine across Ldlr^−/−^, ApoE^−/−^ and C57BL/6 mice, in the absence of intestinal inflammation (Chang et al., 2024).

In this work, we tested our hypothesis that sub-chronic exposure to ultrafine PM induces alterations in the gut microbiota composition of various intestinal regions, in association with systemic prooxidative and proinflammatory effects as well as worsened atherosclerosis. We analyzed gut microbiota composition in several intestinal segments and bacterial metabolites such as SCFAs in the feces of ApoE^−/−^ mice exposed to ultrafine PM *vs.* FA for 10 weeks and studied their correlations with systemic effects and atherosclerotic lesions in the innominate artery and aorta.

## 2. Materials and methods

### 2.1. Animals and exposure protocol

Male ApoE^−/−^ mice (Strain #:002052, B6.129P2-*Apoe*^*tm1Unc*^/J) (5–7 weeks old) were purchased from The Jackson Laboratory (Bar Harbor, Me) and housed in autoclaved cages with cornhusk bedding and allowed access to autoclaved water and standard rodent chow (#5053, Lab Diets, St. Louis, MO) *ad libitum* during non-exposure periods with a 12-h light/dark cycle. Inhalation exposures were conducted as previously described (Chang et al., 2024). Briefly, following at least 1 week of acclimation, ApoE^−/−^ mice (8 weeks old) were transferred to exposure chambers and subjected to exposures to re-aerosolized PM, highly enriched in particles in the ultrafine-size range (ultrafine PM) or filtered air (FA) for 6 h/day (9:00 AM to 3:00 PM), 3 days/week for 10 weeks. Aqueous slurries of ambient PM samples were re-aerosolized into the ultrafine size range using a Hope nebulizer (B&B Medical Technologies, USA), aiming to obtain an aerosol with particles smaller than 0.2 μm with HEPA-filtered air flow as previously described (Taghvaee et al., 2019). Animals exposed to FA were subjected to HEPA-filtered air during the exposures which ensured almost complete particulate-free air with 99.97 % filter retention of 0.3 μm Dispersed Oil Particulate (DOP) aerosol (PALL Life Sciences). All mice in both FA and ultrafine PM-exposed groups were subjected to identical conditions between exposure sessions (temperature: 72-73°F and relative humidity: 30–70 %) including during non-exposure days. Mice were housed in an environment in which they received filtered air through Minimum Efficiency Reporting Values (MERV 15) filters that resulted in an average filtering efficiency ≥95 % for particles 3.0–10.0 μm, ≥ 90 % for 1.0–3.0 μm, and ≥85 % for 0.3–1.0 μm particles. Our research protocol was conducted in compliance with the Animal Research Committee and Institutional Animal Care and Use Committee (IACUC) at the University of California, Los Angeles (UCLA).

### 2.2. PM collection and characterization

Ambient PM_2.5_ was collected on PTFE membrane filters (20 × 25 cm, 3.0 μm pore size, PALL Life Sciences, USA) using a high-volume sampler (Misra et al., 2002) operating at a sampling flow rate of 400 lpm between February-May 2019, for conducting mouse exposures in the ultrafine size range that took place between August-November 2019. Samples were collected throughout the year at our central monitoring site at USC. The “USC site” is located near downtown Los Angeles and it is about 150 m downwind of the CA-110 freeway. This is the central site that represents a typical urban pollution mix in downtown Los Angeles. The site is rich in freshly emitted UFP. In the wintertime, UFP at this site has a high Semi Volatile Organic Compound (SVOC) content, whereas in the summertime, this site is also impacted by secondary processes (Fine et al., 2004; Ning et al., 2007). Further details regarding the PM collection, extraction and characterization have been previously reported by us (Chang et al., 2024; Soleimanian et al., 2020; Taghvaee et al., 2019).

### 2.3. Sample preparation and 16S rRNA gene sequencing

Following euthanasia, intestines were harvested and divided by segments for collection of mucosal and luminal samples from cecum, jejunum and ileum as described previously (Chang et al., 2024; Jacobs et al., 2017). Further details regarding the preparation of mucosal and luminal intestinal samples for DNA extraction and 16S ribosomal RNA gene sequencing have been previously described by us (Chang et al., 2024).

### 2.4. Microbiome diversity analysis

Details regarding the assessment of alpha and beta microbiome diversity using the Shannon index and Bray-Curtis dissimilarity matrix, respectively, have been previously described by us (Chang et al., 2024; Jacobs et al., 2016). In brief, significance of α-diversity differences were assessed using Kruskal Wallis rank sum test in R, where exposure group was considered a fixed effect and cage as a random effect. Data were filtered to exclude samples with less than 10,000 reads for β-diversity and differential abundance analysis. Statistical analyses for β-diversity was conducted using permutational multivariate analysis of variance (PERMANOVA) implemented in the Adonis package in R with treatment as a fixed effect and cage as strata for permutations. Differences in the relative abundances of microbial taxa were analyzed by MaAsLin2 in R (Version 1.4.1106) with treatment as a fixed effect, and cage or mouse ID as a random effect (Mallick et al., 2021). Significantly abundant taxa were visualized using *ggplot* in R Studio.

### 2.5. Atherosclerotic lesion quantification

Atherosclerotic lesion quantification was performed by *en face* as described previously (Li et al., 2013) with minor modifications. Briefly, entire aortas (thoracic and abdominal) with the proximal portions of the head vessels (innominate artery, left carotid artery and left subclavian artery) were dissected after perfusing the liver with 1X PBS and stored in formalin sucrose solution (4 % paraformaldehyde, 7.5 % w/v sucrose, 10 mM Sodium phosphate buffer at pH 7.35, 2 mM EDTA and 20 μM Butylated hydroxytoluene) at 4 °C. The aortas were split longitudinally from ascending aorta to the iliac bifurcation and pinned on a black wax pan for staining with Sudan IV solution (5 g of Sudan IV (Sigma-Aldrich, St. Louis, MO), 500 mL of 70 % ethanol and 500 mL of 100 % acetone). The aortas were washed using 70 % ethanol for 5 min, followed by Sudan IV staining for 15 min and further destained in 80 % ethanol for 2–3 min, and then stored in PBS. The images of the aorta and lesions were captured by using a high-resolution dissection microscope (Toup-Cam^™^ XCAM 4 K UHD 8.0MP) with 40X magnification. Lesion quantifications were performed in the innominate artery, aortic arch and whole aorta, using Image J software (Image J 1.53 k, National Institutes of Health). The quantification of lesions in the whole aorta and aortic arch included the branches (innominate artery, left carotid artery and left subclavian artery) (Orecchioni et al., 2022). The ratios of the area circumscribed by atherosclerotic lesions in the innominate artery, aortic arch and whole aorta, to that of their respective total areas were determined and used as %lesion area scores for conducting correlation analysis.

### 2.6. Lipids and oxidized fatty acids

Total cholesterol, unesterified cholesterol, HDL cholesterol, triglycerides and free fatty acids were determined in the plasma by enzymatic colorimetric assays as per the manufacturer’s instructions (Thermofisher Scientific, Middletown, VA). For liver cholesterol and triglycerides, homogenates of the liver were prepared in NP40 buffer supplemented with Halt^™^ Protease Inhibitor Cocktail (Thermofisher Scientific, Middletown, VA). Concentrations were normalized by total protein levels in the homogenates using the Pierce^™^ BCA Protein Assay Kit as per the manufacturer’s instructions (Thermofisher Scientific, Middletown, VA). Triglycerides in the liver were measured using the Triglyceride Colorimetric Assay Kit (Cayman Chemical, Ann Arbor, MI) and cholesterol levels were measured using the Infinity Cholesterol Reagent by following the manufacturer’s instructions (Thermofisher Scientific, Middletown, VA). For measurement of oxidized fatty acids, lipids were extracted from plasma, liver and bronchoalveolar lavage fluid (BALF), and analyzed by LC-MS/MS as previously described (Yin et al., 2019; Yin et al., 2013).

### 2.7. Lipid peroxidation

Malondialdehyde (MDA) content was measured in mouse liver homogenates using a colorimetric assay kit (Thermofisher Scientific, Middletown, VA). Concentration (nmol/g) of MDA in each sample was calculated using a standard curve. Values that were not obtained within the range of the standard curve were excluded.

### 2.8. BALF collection and cell analysis

BALF was collected from each mouse and analyzed as previously described (Li et al., 2015; Zhang et al., 2008) with slight modifications. Briefly, the left bronchus of each mouse was closed with a suture, and the left lung was harvested. Afterwards, the right lung was lavaged 3 times with 0.5 mL of phosphate buffered saline (PBS) yielding approximately 1.5 mL of BALF per mouse. Total cell counts within the BALF were performed using a hemocytometer under brightfield microscopy. Cell differentials were performed using 200 cells stained with Hema 3^™^ Manual Staining System and Stat Pack (Thermofisher Scientific, Middletown, VA), and scored as particular immune cell types. Total protein was estimated using the Pierce^™^ BCA Protein Assay Kit as per the manufacturer’s instructions (Thermofisher Scientific, Middletown, VA). Lactate dehydrogenase (LDH) content was determined in 50 μL of BALF per sample using CytoTox-ONE homogeneous membrane integrity assay kit (Promega, Madison, WI).

### 2.9. Lung histology

Following euthanasia, a piece of lungs was fixed in a 10 % phosphate-buffered formalin solution and then transferred to 70 % ethanol. The histology cassettes were sent to the Translational Pathology Core Laboratory (TPCL) at UCLA for embedding in paraffin, sectioning (4 μm), and staining with hematoxylin and eosin (H&E) to evaluate the degree of hepatic inflammation. Sections were blindly analyzed by a lung pathologist to assess the extent of immune cell infiltration according to a scoring system described previously (Bayes et al., 2016; Gori et al., 2019) (Table S1). Images of the H&E-stained lung sections were taken using Zeiss Zen Pro V.3.11 software.

### 2.10. Gene expression analysis

Gene expression levels were measured by quantitative PCR (qPCR) as previously described (Chang et al., 2024). Briefly, TRIZOL reagent (Invitrogen, Carlsbad, CA) was used to isolate RNA from tissues, which was followed by cDNA synthesis using the High-Capacity cDNA Reverse Transcription kit (Applied Biosystems, Waltham, MA). qPCR was performed using a LightCycler480 instrument (Roche Molecular Biochemicals) under the following conditions: 95 °C (10 mins), 45 cycles of 95 °C (10 secs), 60 °C (30 secs) and 72 °C (15 secs). Fold change was calculated using 2^−ΔΔCp^ method constructed from the cycle thresholds or crossing point (Cp) values of each dilution sample (Livak & Schmittgen, 2001). qPCR reactions were performed using the TaqMan probes (Thermofisher Scientific, Middletown, VA). Relevant information regarding the TaqMan assay IDs are outlined in Table S2.

### 2.11. Quantification of short chain fatty acids

Mouse fecal samples were extracted and analyzed for SCFAs by direct-injection gas chromatography (GC) at the Center for Human Nutrition in UCLA as described previously (Gupta et al., 2025). Briefly, samples were thawed, weighed and homogenized in acidic distilled H2O (pH 2.0) at 100 mg/mL (w/v), followed by centrifugation at 10,000*g* for 15 min at 4 °C, after which the supernatant was spiked with an internal standard 2-ethylbutyric acid. SCFAs were quantified using gas chromatography flame ionization detection (Agilent 7890A) and StabilWAX-DA column (Restek corp. 30 m × 0.25 mm i.d. 0.25 μm). The Flow rate of Helium supplied as the carrier gas was 1 mL/min. The initial oven temperature was maintained at 95 °C for 30 secs, then raised to 200 °C at 8 °C/min, then increased to 260 °C at 10 °C/min, and was finally held at 260 °C for 5 mins. The temperatures of the flame ionization detector (FID) and the injection port were 240 and 260 °C, respectively. The flow rates of gas including air, nitrogen and hydrogen were 300, 25 and 30 mL/min, respectively. The volume of sample injected for analysis of GC was 1 μL, and each analysis ran for 24.625 min. Individual calibration curves were obtained for each SCFAs by plotting the ratio of peak areas of individual SCFAs to internal standard against the concentration of the individual SCFAs and fit by linear regression. The concentration of SCFAs were calculated based on the calculation curves by using peak area ratio of sample peak against the internal standard.

### 2.12. Paraoxonase activity

Paraoxonase (PON) activity was assessed in plasma samples *via* the rate of hydrolysis of paraoxon substrate (diethyl-*p*-nitro-phenyl phosphate) to *p*-nitrophenol using an assay described previously (Gupta et al., 2021). Briefly, 5 μl of plasma was incubated with the assay mixture that consisted of 2.4 mM paraoxon in 0.1 M Tris-HCl buffer (pH 8.5) containing 2 mM CaCl_2_ and 2 M NaCl at RT. Absorbance was measured at 405 nm every 15 s for 4 min to determine the kinetics of p-nitrophenol formation. PON1 mass concentration was determined in the plasma using the mouse PON1/Paraoxonase1 ELISA Kit PicoKine as per the manufacturer’s instructions (Boster Bio, Pleasanton, CA).

### 2.13. Statistical analysis

Data were reported as mean ± SEM and were analyzed using unpaired Student’s *t* test or Mann Whitney *U* test for comparison of results between 2 groups, depending on whether the data were normally distributed. Associations between the differentially abundant gut microbiome and other metabolic parameters were analyzed using nonparametric linear regression by Spearman’s correlation. All other associations were conducted using parametric linear regression by Pearson’s correlation calculated in GraphPad Prism 10.4.1 software for Windows. All microbiota data were statistically adjusted for cage effects as described above. Statistical tests and number of samples used for each experiment were specified in the figure legends. *p* < 0.05 was considered statistically significant. For microbiome analysis, *p*-values were adjusted for multiple comparisons using the Benjamin–Hochberg procedure and the significance threshold was set at *p.adj* < 0.10.

## 3. Results

### 3.1. Exposure to re-aerosolized PM in the ultrafine-size range promotes atherosclerosis

Male ApoE^−/−^ mice, fed a chow diet, underwent exposures to reaerosolized PM in the ultrafine size-range (ultrafine PM) or FA for 6 h/day, 3 days/week for 10 weeks, as described in the methods. The total particle number concentration (PNC) in the exposure aerosol was 367,356 particles/cm^3^, with 95 % of the particles under 0.1 μm in diameter (0.013–0.76 μm), and almost all of them below 0.2 μm (Fig. S1), a mode and median diameter of 51.4 nm and 57.3 nm, respectively, and a target average mass concentration of 300–350 μg/m^3^, as previously reported (Chang et al., 2024). The chemical characterization and composition analysis of PM aerosols shown in Table S3 indicates that the greatest fraction of PM was total carbon (~48 %).

**Fig. 1.**
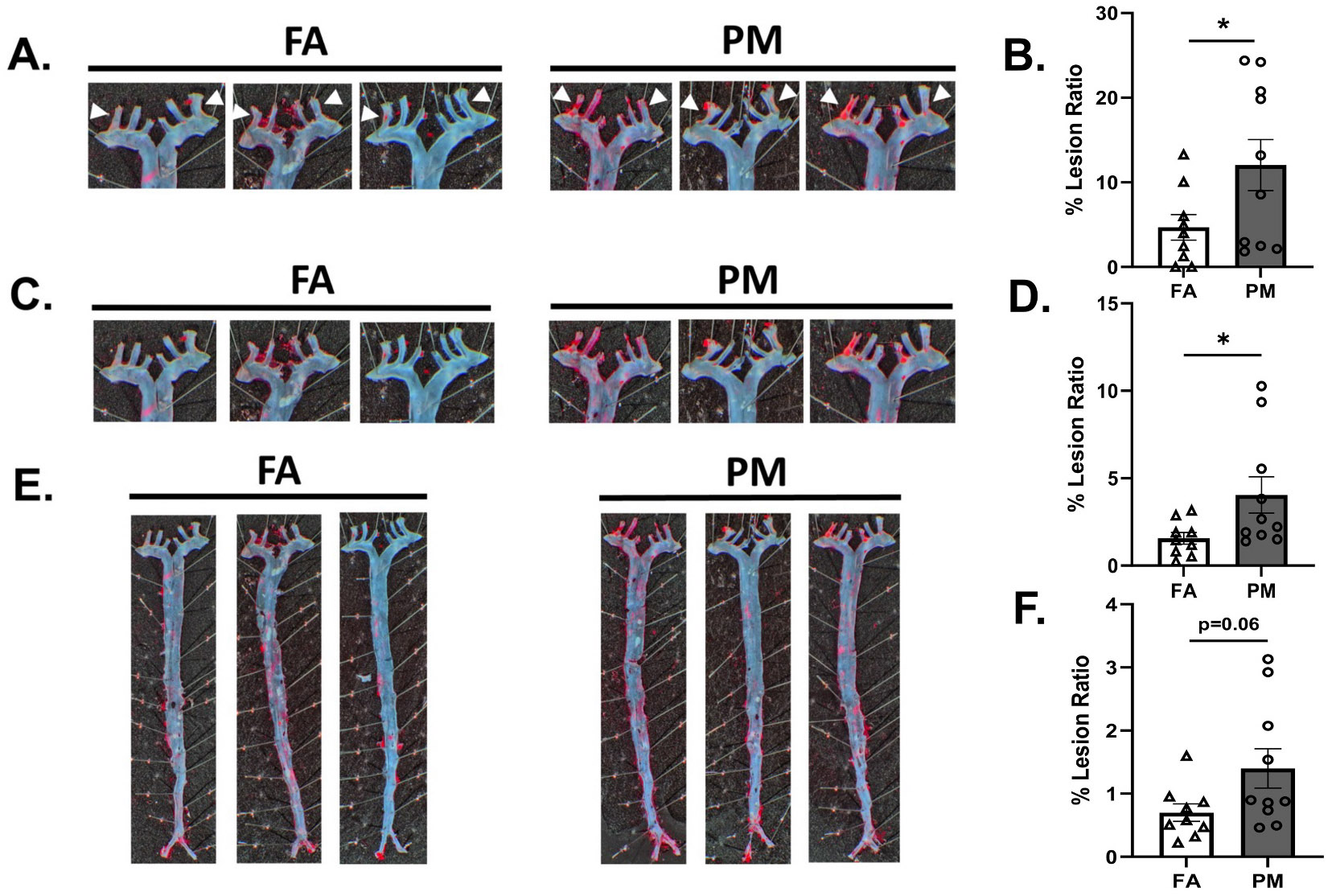
Ultrafine PM exposure and atherosclerosis. Representative images of aorta stained with Sudan IV to assess atherosclerotic lesions in (A) innominate artery (indicated with white arrows), (C) aortic arch, and (E) whole aorta of ultrafine PM *vs.* FA exposed mice. %Lesion ratios were quantified using ImageJ and shown in panels (B), (D) and (F) for innominate artery, aortic arch and whole aorta, respectively. Each bar denotes mean ± SEM (n = 9 (FA) and n = 10 (PM)). One sample each from the FA and PM group was excluded due to technical concerns with Sudan IV staining. Statistical significance between the two groups were determined using Student’s *t*-test with Welch’s correction. *p < 0.05, ultrafine PM *vs.* FA.

Mice exposed to ultrafine PM for 10 weeks exhibited a significant 158 % increase in atherosclerotic lesions in the innominate artery (p = 0.048, Fig. 1A-B), 158 % in the aortic arch (p = 0.045, Fig. 1C-D) and almost significant 100 % increase in the whole aorta (p = 0.062, Fig. 1E-F) in comparison to FA-exposed mice, demonstrating the pro-atherogenic potential of ultrafine particulates and consistent with our previous reports (Araujo et al., 2008; Li et al., 2013). On the other hand, there were no significant differences in the levels of plasma or hepatic lipids between the ultrafine PM and FA-exposed mice (Table 1).

We evaluated effects of ultrafine PM exposure on the lungs, and observed no significant differences in total cell counts, cell differentials, total protein or lactate dehydrogenase (LDH) levels in the BALF of ultrafine PM *vs.* FA-exposed mice (Fig. S2). Likewise, there were no differences in the BALF levels of hydroxyeicosatetraenoic acids (HETEs) and hydroxyoctadecadienoic acids (HODEs), which are oxidized metabolites of arachidonic acid (AA) and linoleic acid (LA), respectively (Fig. S3A-B and Table S4) together with no changes in the mRNA levels of proinflammatory cytokine expression in the lungs (Fig. S3C). Furthermore, histological assessment of hematoxylin and eosin (H&E)-stained lung sections showed retention of the normal alveolar architecture, without significant acute or chronic interstitial inflammatory infiltrates in either group (Fig. S4), and a histological score of 0 as assessed blindly by a pathologist using the histological scoring criteria by Bayes et al (Bayes et al., 2016) outlined in Table S1.

### 3.2. Ultrafine PM altered the gut microbial composition in correlation with increased atherosclerotic lesions

**Fig. 2.**
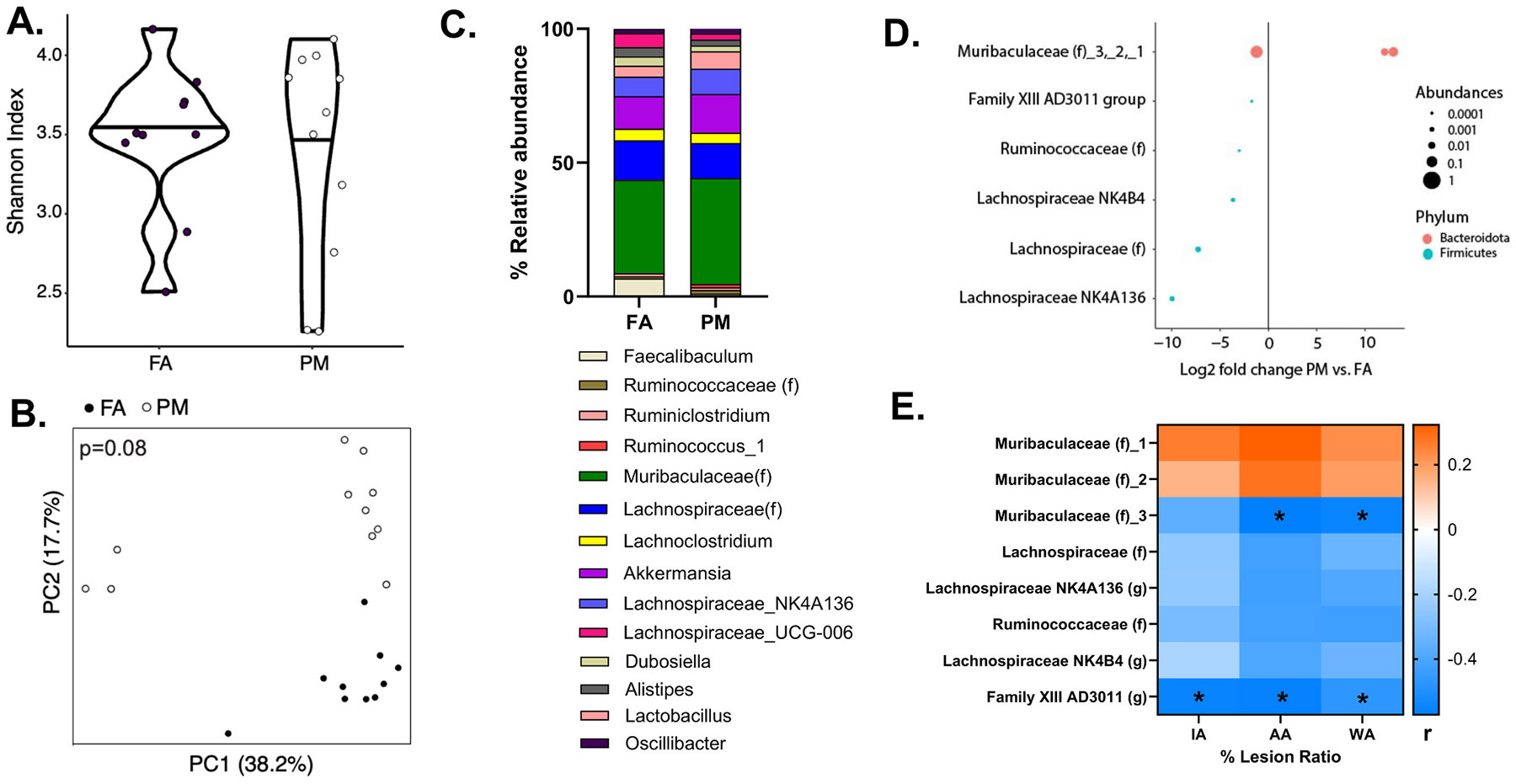
Ultrafine PM exposure and luminal cecal microbiota composition. (A) α-diversity using Shannon index. Statistical significance was determined by a linear mixed effects model with adjustment for cage effects (p = 0.89). (B) β-diversity, by principal coordinates analysis (PCoA) of Bray-Curtis dissimilarity. Significance was assessed by permutational multivariate analysis of variance (PERMANOVA) with *p* value as indicated above. (C) Relative abundance of microbial composition at the genus level. (D) Log2 fold change of microbiota abundance between the ultrafine PM and FA exposed mice. Dot size is proportional to the differential microbiota abundance. (E) Heat maps representing Spearman’s correlations between the relative abundance of microbiota with %lesion ratio in the innominate artery (IA), aortic arch (AA) and whole aorta (WA) of ultrafine PM and FA exposed mice. Orange colors indicate positive associations, whereas blue colors indicate negative associations based on the correlation coefficient (r) as indicated above. All the microbial taxa shown in the heat map correspond to the differentially abundant taxa presented in Panel (D). Various microbial taxa within the same *Muribaculaceae (f)* are indicated by “_1,_2,_3”. Significant associations are denoted by asterisks. *p < 0.05, n = 10 (FA) and n = 11 (PM).

**Fig. 3.**
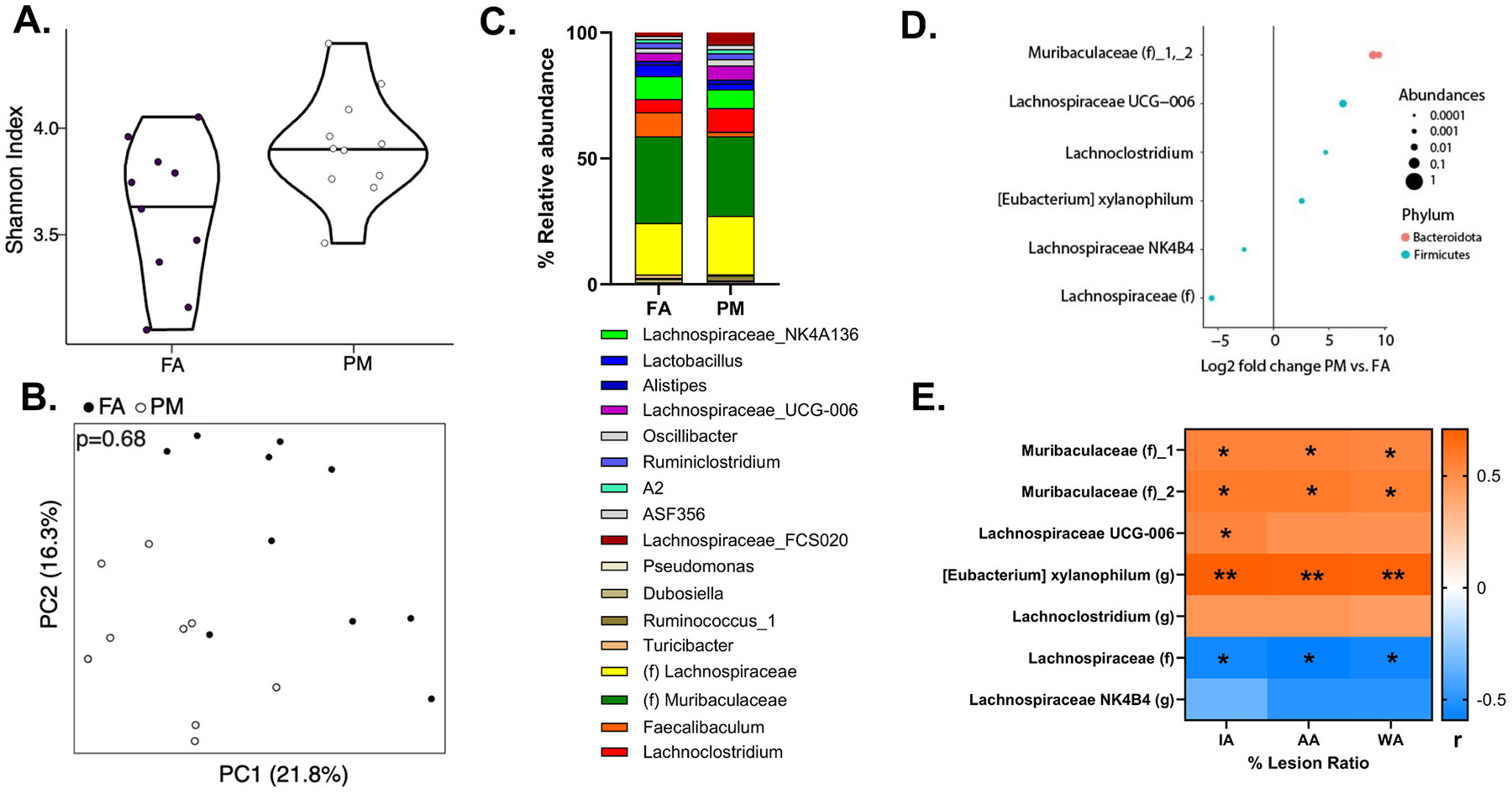
Ultrafine PM exposure and mucosal cecal microbiota composition. (A) α-diversity using Shannon index. Statistical significance was determined by a linear mixed effects model with adjustment for cage effects (p = 0.23). (B) β-diversity, by principal coordinates analysis (PCoA) of Bray-Curtis dissimilarity. Significance was assessed by permutational multivariate analysis of variance (PERMANOVA) with *p* value as indicated above. (C) Relative abundance of microbial composition at the genus level. (D) Log2 fold change of microbiota abundance between the ultrafine PM and FA exposed mice. Dot size is proportional to the differential microbiota abundance. (E) Heat maps representing Spearman’s correlations between the relative abundance of microbiota with %lesion ratio in the innominate artery (IA), aortic arch (AA) and whole aorta (WA). Orange colors indicate positive associations, whereas blue colors indicate negative associations based on the correlation coefficient (r) as indicated above. All the microbial taxa shown in the heat map correspond to the differentially abundant taxa presented in Panel (D). Various microbial taxa within the same *Muribaculaceae (f)* are indicated by “_1,_2”. Significant associations are denoted by asterisks. *p < 0.05 and **p < 0.01, n = 10 (FA) and n = 11 (PM), except for panels (B) and (D), where n = 10 (FA) and n = 9 (PM), since β-diversity and differential abundance data underwent minimum depth filtering to remove samples with low read counts to help normalize the data, as described in the methods. Two samples showed the lowest sample depths of 6,588 and 7,662. Since minimum depth filtering was performed (e.g. > 10,000), those two samples from the ultrafine PM group were excluded from this analysis.

We characterized changes in the microbial composition in the cecum by 16S rRNA gene sequencing. Microbial diversity within and between groups was assessed through α- and β-diversity indices. While there were no significant differences in luminal cecal microbial diversity by Shannon index (p = 0.89, Fig. 2A), there was a borderline significant trend using Bray-Curtis dissimilarity (p = 0.08, Fig. 2B), after adjustment for cage effects, as described in the methods. Importantly, differential abundance analysis demonstrated significant shifts in several microbial taxa in mice exposed to ultrafine PM *vs.* FA (Fig. 2C) as evidenced by statistically significant alterations in several microbial taxa, shown by log2 fold change values in Fig. 2D. We further conducted Spearman’s correlation analysis to determine whether changes in the gut microbiota composition were associated with changes in atherosclerotic lesions. Importantly, there were significant negative correlations between altered luminal cecal microbiota composition and percentage of lesion ratio in the innominate artery, aortic arch and whole aorta, as shown in the heat map (Fig. 2E). Likewise, there were no significant differences in mucosal cecal microbial diversity by Shannon index (p = 0.23, Fig. 3A) or Bray-Curtis dissimilarity (p = 0.68, Fig. 3B) but differential abundance analysis demonstrated shifts in several microbial taxa in mice (Fig. 3C) as well, some of which were significantly altered in the mucosal cecum of ultrafine PM *vs.* FA-exposed mice (Fig. 3D) with significant associations between altered mucosal cecal microbiota composition and percentage of lesion ratio in the innominate, aortic arch and whole aorta (Fig. 3E). These alterations in microbial composition add to changes observed in the microbiome of feces and small intestine (mucosal jejunum and mucosal ileum) as previously reported by us (Chang et al., 2024). However, while there were significant correlations between changes in mucosal and luminal cecal microbiota with atherosclerosis, there were none with the microbiota composition in the mucosal and luminal jejunum, mucosal ileum or feces obtained after 10 weeks of PM exposure (data not shown).

### 3.3. Ultrafine PM enhanced fecal short chain fatty acid levels correlating with changes in atherosclerosis and cecal microbiota composition

**Fig. 4.**
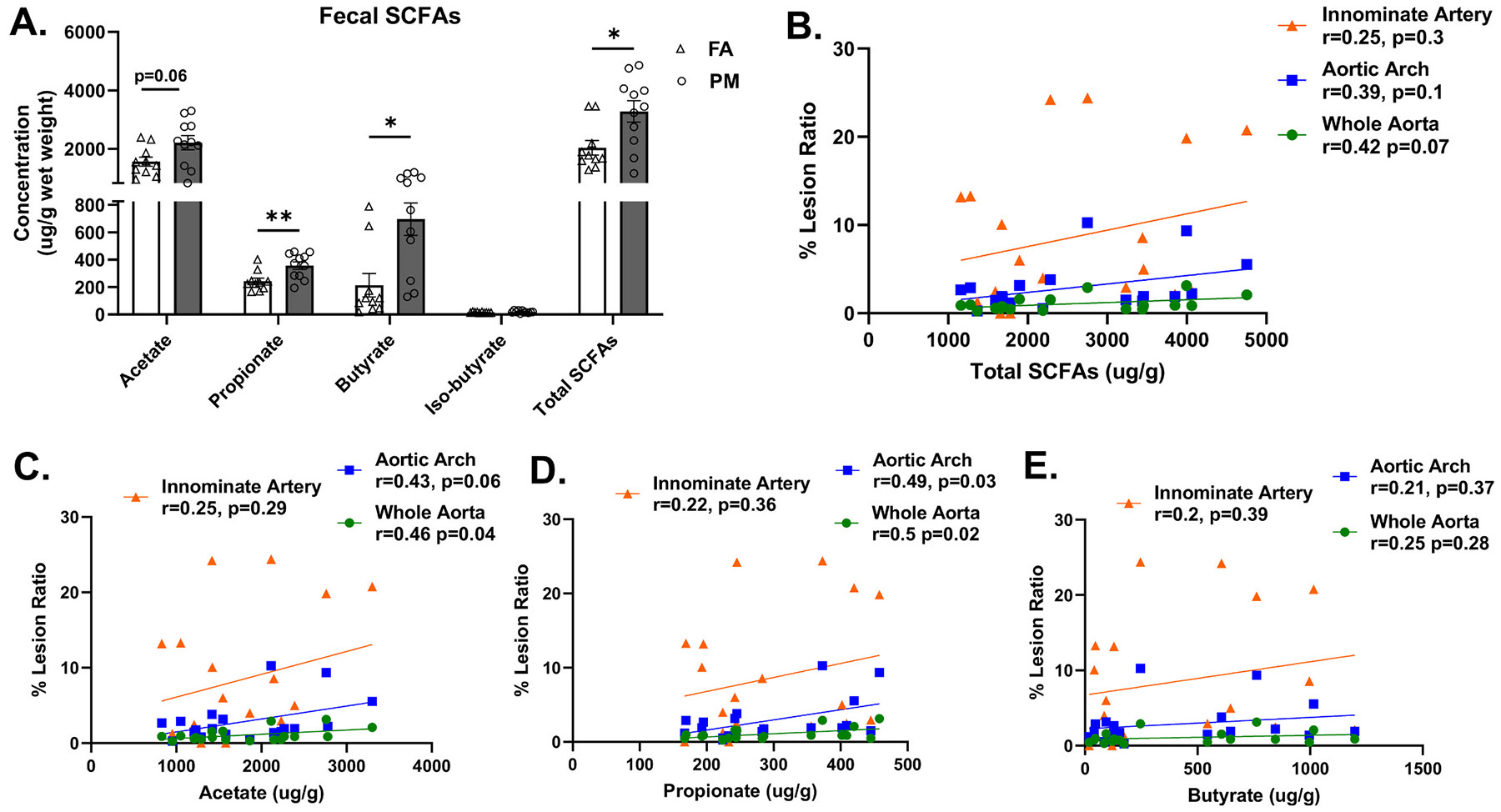
Fecal levels of short chain fatty acids and their association with atherosclerotic lesions. (A) Fecal levels of short chain fatty acids (SCFAs), including acetate, propionate, butyrate, *iso*-butyrate and their sum (total SCFAs) in ultrafine PM and FA-exposed mice. Each bar denotes mean ± SEM (n = 10 (FA) and n = 11 (PM)). *p < 0.05 and **p < 0.01, using Student’s *t*-test. Pearson’s correlation analysis between fecal levels of (B) total SCFAs, (C) Acetate, (D) Propionate and (E) Butyrate, and atherosclerotic lesions (% lesion ratio) in the innominate artery, aortic arch and whole aorta of ultrafine PM and FA-exposed mice. *p* values and correlation coefficients (r) are as shown.

We asked whether ultrafine PM-induced changes in the gut microbiome were accompanied by changes in bacterial metabolites such as SCFAs. Interestingly, ultrafine PM exposures led to marked increases in the levels of acetate, propionate, butyrate, and the sum of all SCFAs (Fig. 4A) in comparison to FA, with either borderline significant association between total fecal SCFAs and %lesion ratio in the whole aorta (r = 0.42, p = 0.07, Fig. 4B), or significant positive association between acetate and %lesion ratio in the whole aorta (r = 0.46, p = 0.04, Fig. 4C), propionate and whole aorta (r = 0.5, p = 0.02) as well as aortic arch (r = 0.49, p = 0.03, Fig. 4D), but no significant associations involving butyrate (Fig. 4E). Furthermore, changes in other differentially abundant microbial taxa in the luminal and mucosal cecum also associated either positively (orange colors) or negatively (blue colors) with fecal SCFAs, as represented by the heat maps (Fig. S5). Thus, our data indicates that ultrafine PM-induced alterations in cecal microbial composition resulted in elevated SCFAs, which correlated with worsened atherosclerosis as well as specific changes in cecal microbial taxa.

### 3.4. Ultrafine PM induced systemic prooxidative effects correlating with changes in cecal microbiota composition and worsened atherosclerosis

To determine whether changes in the cecal microbiota composition correlated with cardiometabolic effects induced by ultrafine PM previously observed by us (Li et al., 2013), we assessed the functionality of paraoxonase 1 (PON1) in plasma, which is an antioxidant enzyme known to contribute to HDL-mediated antioxidant protection (Mackness et al., 1993), that we have previously reported to be inhibited after ambient PM (Li et al., 2013; Lin et al., 2019) or Diesel exhaust exposures (Yin et al., 2013). Mice exposed to ultrafine PM exhibited a trend towards reduced PON activity in plasma (p = 0.11) (Fig. S6A), without significant changes in its mass concentration in plasma either (Fig. S6B). Interestingly, PON activity but not PON1 mass not only associated negatively with changes in the differentially abundant mucosal cecal microbial taxa including *Muribaculaceae (f)* [r = −0.58, p = 0.02], *Lachnospiraceae UCG-006* [r = −0.57, p = 0.02] and *[Eubacterium] xylanophilum (g)* [r = −0.58, p = 0.02], but also with %lesion ratio in the aortic arch (r = −0.48, p = 0.03) and whole aorta (r = −0.4, p = 0.08) (Fig. S6C-D).

**Fig. 5.**
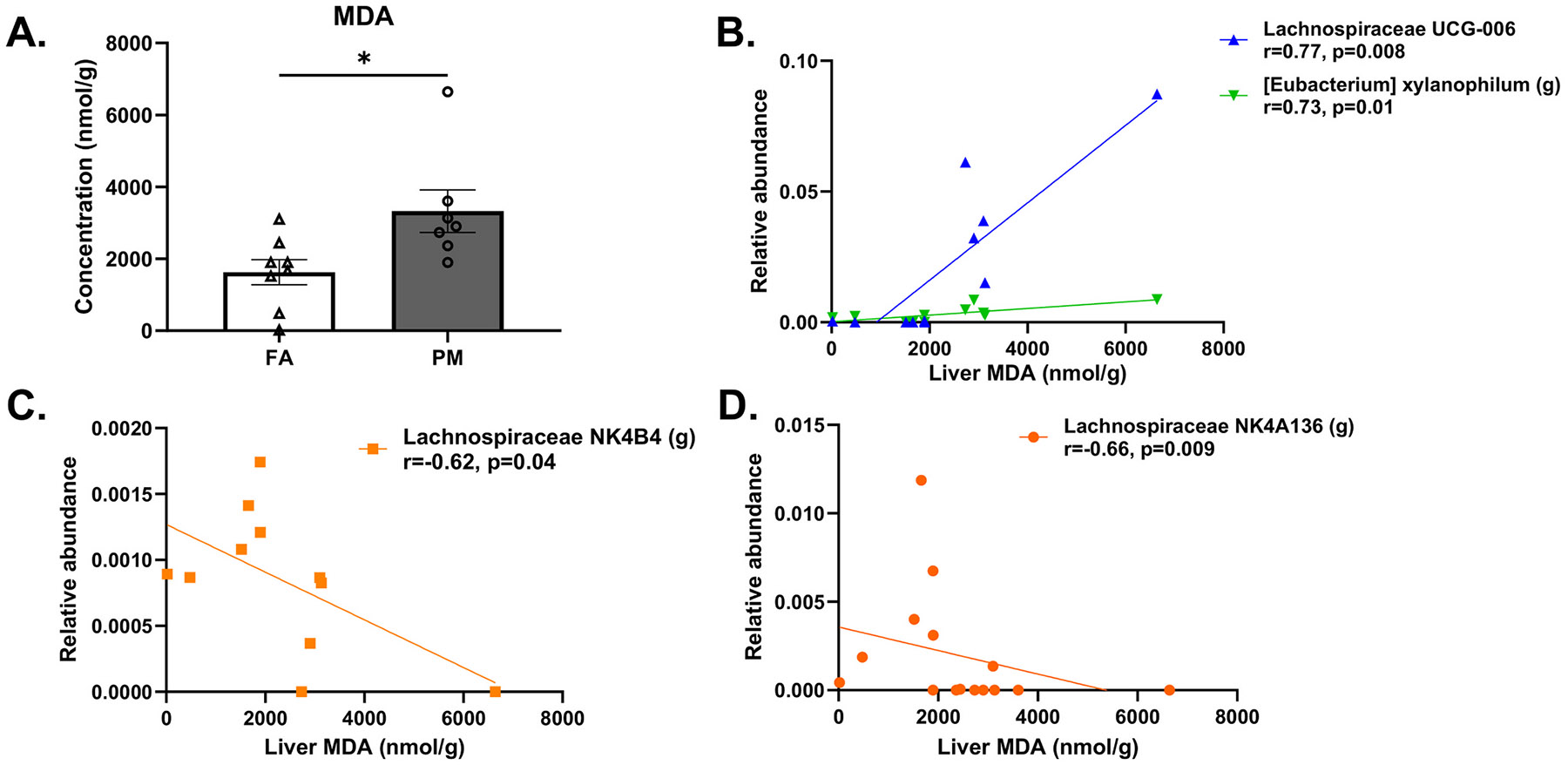
Ultrafine PM induced liver lipid peroxidation in association with gut microbiota dysbiosis. (A) Hepatic levels of MDA were determined in the liver homogenates as described in the materials and methods. Each bar denotes mean ± SEM (n = 8 (FA) and n = 7 (PM)). Statistical significance between the two treatment groups were determined using Mann Whitney’s *U* test. Spearman’s correlation analysis indicating significant associations between liver MDA levels and relative abundance of mucosal cecal microbial taxa (B and C) that are either positive (B) or negative (C). (D) Associations between liver MDA levels and luminal cecal microbial taxa of ultrafine PM and FA exposed mice. *p* values and correlation coefficients (r) are shown above. *p < 0.05, ultrafine PM *vs*. FA. MDA; malondialdehyde.

**Fig. 6.**
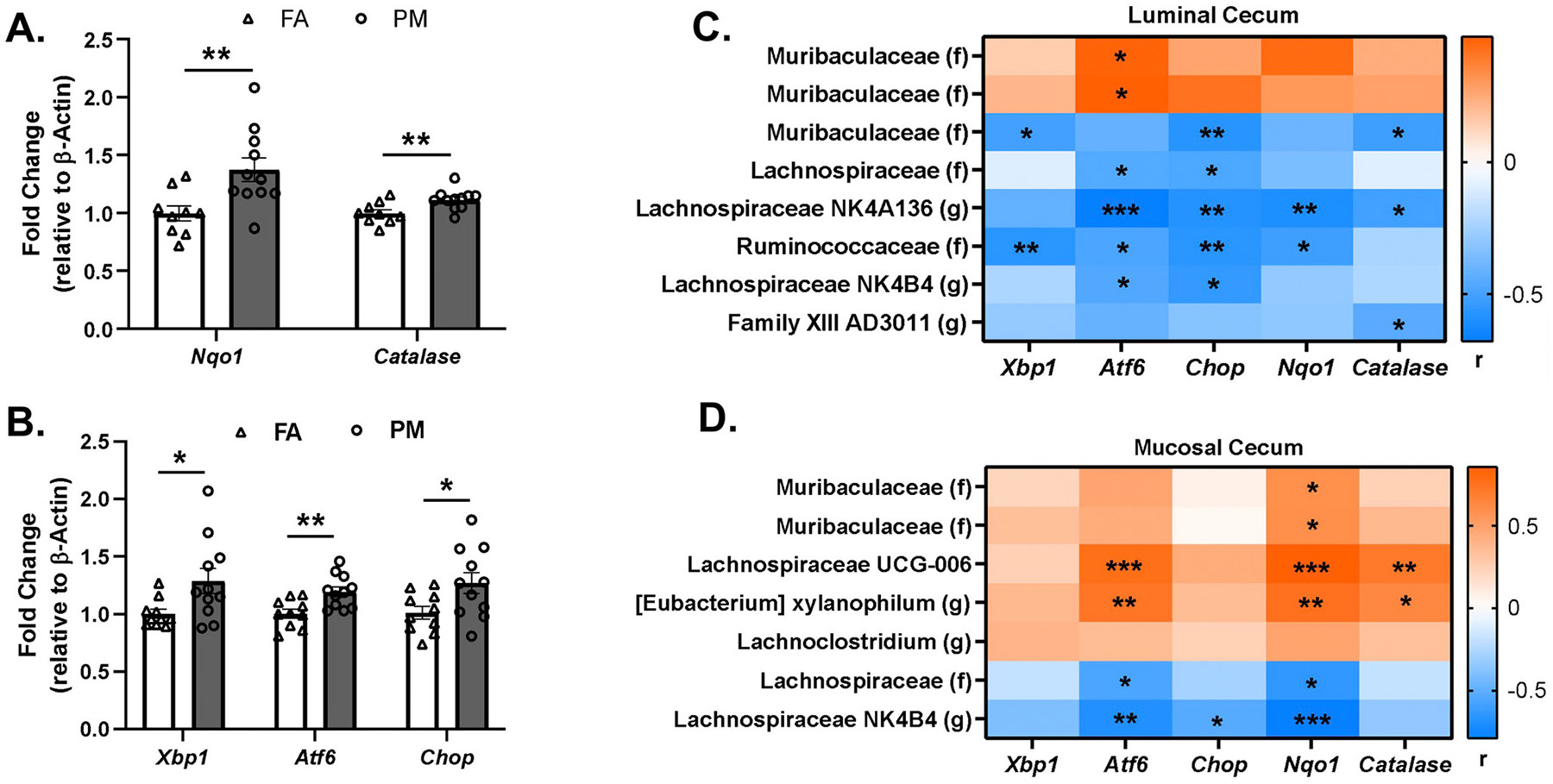
Ultrafine PM induced ER and oxidative stress in the liver in association with gut microbiota dysbiosis. Hepatic mRNA levels expressed as fold change of (A) antioxidant genes and (B) endoplasmic reticulum (ER) stress genes as determined by qPCR. Each bar denotes mean ± SEM (n = 9 (FA) and n = 11 (PM) for panel (A) and n = 10 (FA) and n = 11 (PM) for panel (B)). Statistical significance between the two treatment groups were determined using Student’s *t* test. Heat maps representing Spearman’s correlation analysis between the relative abundance of (C) luminal and (D) mucosal cecal microbiota with ER stress and antioxidant gene expression levels. Orange colors indicate positive associations, whereas blue colors indicate negative associations based on the correlation coefficient (r) as indicated above. All the microbial taxa shown in the heat maps for luminal and mucosal cecal microbiome correspond to the differentially abundant taxa. Significant associations are denoted by asterisks. *p < 0.05, **p < 0.01 and ***p < 0.001, ultrafine PM *vs.* FA.

We then investigated whether ultrafine PM led to oxidative stress in the liver through assessment of lipid peroxidation and induction of ER stress, and observed statistically significant elevations in malondialdehyde (MDA) content in the livers of ultrafine PM *vs*. FA-exposed mice (Fig. 5A), which were consistent with our previous studies (Araujo et al., 2008). Interestingly, liver MDA levels significantly correlated positively with mucosal cecal microbial taxa including *Lachnospiraceae UCG-006* [r = 0.77, p = 0.008] and *[Eubacterium] xylanophilum (g)* [r = 0.73, p = 0.01] (Fig. 5B), and negatively associated with *Lachnospiraceae NK4B4 (g)* [r = −0.62, p = 0.04] and *Lachnospiraceae NK4A136 (g)* [r = −0.66, p = 0.009] in the mucosal and luminal cecum, respectively (Fig. 5C-D). In addition, ultrafine PM induced a significant upregulation in the hepatic mRNA levels of phase II antioxidant genes such as NAD(P)H quinone oxidoreductase 1 (*Nqo1*) and *Catalase* (Fig. 6A), together with activation of endoplasmic reticulum (ER) stress pathways as evidenced by a significant upregulation in the hepatic mRNA levels of X-box binding protein 1 (*Xbp1*), activating transcription factor 6 (*Atf6*) and C/EBP Homologous Protein (*Chop*) (Fig. 6B), consistent with our previous studies (Araujo et al., 2008; Gong et al., 2007; Yin et al., 2013). On the other hand, levels of all individual HETEs, HODEs, AA, LA (Table S4) as well as total HETEs and HODEs (Fig. S7) remained unaltered either in the plasma or liver of ultrafine PM-exposed mice. In addition, there were no changes in the hepatic mRNA levels of various proinflammatory cytokines including interleukin 1α (*Il-1α*), interleukin 1β (*Il-1β*), interleukin 6 (*Il-6*) and tumor necrosis factor α (*Tnfα*) (Fig. S8). Importantly, however, liver MDA levels were associated with worsened atherosclerosis and various prooxidative effects, as evidenced by a significant correlation between liver MDA content and %lesion ratio in the innominate artery, aortic arch and whole aorta (Fig. S9A), plasma PON activity (Fig. S9B), and mRNA levels of antioxidant and ER stress genes (Fig. S9C-D). Furthermore, there were significant associations between altered microbiota composition in the luminal and mucosal cecum and changes in mRNA levels of antioxidant and ER stress genes, as shown in the heat maps (Fig. 6C-D), as well as significant associations between fecal levels of acetate (r = 0.44, p = 0.05) and butyrate (r = 0.44, p = 0.05) with hepatic mRNA levels of *catalase*, and between fecal levels of propionate and hepatic mRNA levels of *Chop* (r = 0.5, p = 0.02) by Pearson’s correlation analysis (Fig. S10). This data indicates that changes in the gut microbiota composition and its metabolites induced by ultrafine PM correlated with systemic prooxidative effects, that could play a role in atherosclerotic lesion development.

## 4. Discussion

Our study demonstrates that ApoE^−/−^ mice exposed to re-aerosolized PM in the ultrafine-size range by inhalation exhibited altered gut microbiota composition, in association with increased atherosclerotic lesions. Ultrafine PM also enhanced fecal SCFAs and promoted systemic prooxidative effects resulting in elevated MDA content and ER stress in the liver. Importantly, there were significant correlations between altered cecal microbiota composition, fecal SCFAs, systemic prooxidative effects and atherosclerotic lesions.

Exposure to ultrafine PM for 10 weeks, using our particle reaerosolization technology (Li et al., 2013; Morgan et al., 2011), exacerbated atherosclerotic lesion development (Fig. 1), without any effects on plasma or hepatic lipids (Table 1). This was consistent with our previous study in chow-fed ApoE^−/−^ mice exposed to ultrafine concentrated ambient particles (CAPs) for 5 weeks that also resulted in increased aortic atherosclerosis as compared to FA controls, and induced biomarkers of systemic oxidative stress without effects on plasma total or HDL cholesterol, using the Versatile Aerosol Concentration Enrichment System (VACES) technology (Araujo et al., 2008). In that study, there was also a significant increase in atherosclerotic lesions in comparison to PM_2.5_-exposed mice, which occurred in parallel with a greater number of sub-0.18 μm particles, and in spite of a markedly smaller exposure PM mass (Araujo et al., 2008). In the current study, mice were subjected to 10 weeks of intermittent exposure to 300–350 μg/m^3^ PM concentration, which were a reasonable approximation to human exposures. After adjusting for differences in weight, minute ventilation and lifetime between mice and humans, the PM concentration that would result in humans in the same PM dose per kilogram body weight that mice were exposed to, would be ~ 40–45 μg/m^3^, which is typical of human exposures to PM_2.5_ in Los Angeles, as well as in other urban areas in California (Hasheminassab et al., 2014). This is also consistent with our previous study where Ldlr^−/−^ mice fed a high fat diet were exposed to similar concentration of ultrafine PM for 10 weeks resulting in an increase in atherosclerotic lesions as well (Li et al., 2013).

Ultrafine PM exposure also altered the microbiota composition in the luminal (Fig. 2C-D) and mucosal cecum (Fig. 3C-D), even after adjustment for cage effects (Laukens et al., 2016), despite no significant differences in α- or β-diversity indices. In general, there was concordance in microbiome changes in luminal and mucosal cecum. However, there were many more differences with the fecal microbiome with the exception of *Lachnospiraceae (f)* and *Ruminococcaceae (f)* which were significantly reduced in the luminal cecum (Fig. 2D) as well as in the feces (Chang et al., 2024). Likewise, we recently reported alterations in the gut microbiome of chow-fed ApoE^−/−^ mice after 16-week inhalation of whole DE, enriched in ultrafine particles, which exhibited significant decreases in *Ruminococcaceae_UCG0.013* and *Ruminococcaceae_UCG0.014* in the cecum as well (Gupta et al., 2025). Changes in microbiome have also been reported after inhalation of PM_2.5_ (Wang et al., 2018) as well as oral administration of ambient ultrafine PM (Li et al., 2017) or DE (van den Brule et al., 2021).

Importantly, changes in the differentially abundant cecal microbiota significantly associated with atherosclerotic lesions in the innominate artery and aorta (Figs. 2E and 3E), with the strongest positive associations observed with *[Eubacterium] xylanophilum (g)*, which also positively associated with liver MDA (Fig. 5B). However, the abundance of *Eubacterium* has previously been shown to be significantly lower in patients with carotid atherosclerosis as compared to healthy controls (Karlsson et al., 2012), which suggests a protective role against atherosclerosis progression instead. Interestingly, differentially abundant microbiota in the mucosal and luminal jejunum, mucosal ileum and feces obtained after 10 weeks of PM exposure, did not associate with atherosclerotic lesions (data not shown), suggesting that changes in cecal microbiota are likely to exert a bigger impact on atherogenesis than in the other intestinal segments.

It is unclear, however, how inhaled PM could alter the gut microbiome. It has been postulated that particles or their chemical constituents could enter the GI tract after being scavenged by alveolar macrophages upon inhalation, and then get ingested following mucociliary clearance (Kreyling et al., 1999). Particles could have also deposited on the fur of mice and have gained access to the GI tract *via* oral ingestion. Changes in the gut microbiome could not be due to intestinal inflammation as evidenced by the lack of histological evidence for immune cell infiltration or upregulation of proinflammatory cytokine expression in the gut (Chang et al., 2024), indicating the involvement of other mechanisms, which will need to be determined in the future.

Ultrafine PM exposure enhanced hepatic MDA levels (Fig. 5A) as well as mRNA expression of phase-II antioxidant and ER stress response genes in the liver (Fig. 6A-B), a response that typically involves intracellular signaling pathways aiming to protect cells from the buildup of misfolded proteins (Ron & Walter, 2007). These results are consistent with our previous reports of increased lipid peroxidation as well as upregulation of antioxidant genes and unfolded protein response (UPR) transcription factors in the liver of ApoE^−/−^ mice exposed to ultrafine CAPs (Araujo et al., 2008; Gong et al., 2007). Another study also showed that exposure to PM_2.5_ activated ER stress-induced apoptosis in the lungs and liver of mice (Laing et al., 2010). Interestingly, enhanced liver MDA levels, and antioxidant and ER stress genes significantly correlated with changes in the cecal microbiota composition (Figs. 5B-D and 6C-D), suggesting the potential interactions between the gut microbiome with effects and responses in the liver, that could influence disease outcomes (Semenova et al., 2024). Thus, hepatic ER stress has been linked to atherosclerosis as its suppression inhibited atherosclerosis development in ApoE^−/−^ mice, although likely due to improved lipid metabolism (Guan et al., 2016), which was not perturbed in our study (Table 1). While ER Stress in the liver may conduce to the activation of inflammatory pathways (Zhang et al., 2016), we could not detect proinflammatory effects induced by PM in the liver (Fig. S8). Therefore, it remains to be determined whether PM induction of ER stress in the liver could have played a role in its promotion of atherosclerosis.

Ultrafine PM exhibited a trend towards reduced plasma paraoxonase (PON) activity (p = 0.11) (Fig. S6A), likely due to functional changes in PON1, an HDL-associated enzyme that harbors anti-atherogenic properties and protects against macrophage-mediated LDL oxidation (Efrat & Aviram, 2010). Indeed, we have reported PON1 activity reduction together with development of prooxidant and/or proinflammatory HDL in ApoE^−/−^ mice exposed to DE for 2 weeks (Yin et al., 2013), as well as in Ldlr^−/−^ mice exposed to ultrafine PM for 10 weeks (Li et al., 2013). It is possible that the decrease in activity was not statistically significant due to compensatory antioxidant responses which could have buffered ultrafine PM effects on PON activity in a similar manner how no changes were observed on plasma or hepatic levels of oxidized fatty acids (Fig. S7 and Table S4). However, it is also possible that significant PON1 inhibitory effects could have been present at earlier time points, in a similar manner how ApoE^−/−^ mice exposed to whole DE for only 2 weeks exhibited PON1 inhibition and increased plasma levels of HETEs and HODEs (Yin et al., 2013). We noticed that PON activity negatively correlated with the %lesion ratio in the whole aorta (p = 0.08) and aortic arch (p = 0.03) (Fig. S6D), which suggests that it may be a factor in atherosclerosis development, but not a main driver, especially since there were no significant differences in between the FA and PM-exposed groups. Interestingly, plasma PON activity negatively correlated with mucosal cecal microbiome (Fig. S6C), which in turn significantly correlated with % atherosclerotic lesions in the aorta (Fig. 3E) as well, suggesting the potential involvement of similar bacterial species in contributing to these endpoints. While genetic manipulation of PON1 has been shown to alter the intestinal microenvironment and microbiota composition (Li et al., 2024), our data suggests the possibility that changes in the gut microbiome could alter PON activity, which will need to be addressed by future studies.

We also observed increased fecal SCFAs in PM-exposed mice (Fig. 4A), some of which correlated positively with the %lesion ratio in the aorta (Fig. 4C-D), as well as mRNA levels of *Xbp1* and *Chop* (Fig. S10). Another study has shown, however, that administration of butyrate or propionate alleviated ER stress, together with reduced coronary microvascular dysfunction (Hong et al., 2024), suggesting a protective role for those SCFAs against ER stress. SCFAs are gut-derived metabolites produced from the bacterial fermentation of dietary fiber in the intestinal tract and are crucial in ameliorating effects on atherosclerosis (Aguilar et al., 2014; Haghikia et al., 2022). Consistent with our data, another study also reported increased fecal SCFAs in C57BL/6 mice exposed to diesel exhaust particles (DEP) *via* intranasal instillation every 2 days for 2 months (Liu et al., 2024). On the contrary, other studies have reported a reduction in SCFAs instead, after inhalation of whole DE (Gupta et al., 2025) or gasoline exhaust (Li et al., 2020) as well as oral administration of DEP (van den Brule et al., 2021) or PM10 (Kish et al., 2013), which could be due to differences in the experimental protocols, including the type of PM and/or mouse models employed. It is known that SCFAs production in the gut is mainly carried out by *Faecalibacterium prausnitzii*, *Ruminococcus*, *Prevotella*, *Bacteroides*, *Bifidobacterium*, *Akkermansia muciniphilia*, and certain species of *Ruminococcaceae* and *Lachnospiraceae* (Parada Venegas et al., 2019; Rey et al., 2010). Indeed, we observed significant enrichment in *Lachnospiraceae UCG.006* and *Ruminococcaceae (f)* in the mucosal cecum (Fig. 3) and mucosal ileum (Chang et al., 2024), respectively, but a reduction in other SCFAs producers including *Ruminococcaceae (f)*, *Lachnospiraceae (f)* and *Lachnospiraceae NK4B4* in the luminal and mucosal cecum (Figs. 2 and 3), as well as in the mucosal ileum, mucosal jejunum and feces of PM-exposed mice (Chang et al., 2024). It is possible then that enrichment in some SCFAs-producing taxa outweighed the reduction in others resulting in a net overall increase. Considering the known anti-inflammatory role of SCFAs (Haghikia et al., 2022), it is less likely that an increase in their levels could have contributed to worsened atherosclerosis either directly or through host receptor mechanisms, including the activation of G-protein-coupled receptors of SCFAs, GPR41 and GPR43 (Wang et al., 2025), which should be addressed by future studies.

We tested whether ultrafine PM exposure induced proinflammatory effects in the lungs that could have mediated the increased atherosclerosis, either by direct interaction *via* the triggering of prooxidative effects or *via* the alterations in the gut microbiota composition which have been reported to lead to the activation of immune responses and disease development in the lungs (Zhou et al., 2021). In our study, no obvious inflammatory effects were detected in the lung (Fig. S2 and S3), as evidenced by normal alveolar architecture and lack of interstitial acute or chronic inflammation (Fig. S4), consistent with our previous studies and the study of Tong et al, where ambient ultrafine PM exposure by inhalation (Araujo et al., 2008; Li et al., 2015) or intratracheal administration (Tong et al., 2010) exhibited systemic effects in the absence of pulmonary inflammation. On the contrary, other studies have shown that two-month continuous exposure to ambient PM_2.5_ (Chen et al., 2013; Wan et al., 2014) or 21-day consecutive inhalation exposure to petrochemical combustion-derived butadiene soot ultrafine PM (Noel et al., 2016) promoted pulmonary inflammation in mice as evidenced by elevated proinflammatory cytokines in the lungs. In addition, we did not observe evidence for prooxidative effects as there were no significant elevations in the levels of HETEs and HODEs in the BALF (Fig. S3A-B and Table S4), unlike our previous study with ApoE^−/−^ mice exposed to whole DE for 2 weeks which showed elevated BALF levels of 12-HETE, 15-HETE and 13-HODE (Yin et al., 2013) or the study of Kampfrath et al where exposure to PM_2.5_ for 20 weeks increased oxidized phospholipids in the BALF (Kampfrath et al., 2011). While we cannot rule out the possibility that these effects might have been induced at earlier timepoints, then triggering compensatory antioxidant homeostatic responses, we could not identify in the lung, obvious mediators responsible for the increased atherosclerosis.

To examine whether changes in the gut microbiome could have mediated PM-induced atherosclerosis, we conducted mediation analysis with bootstrapping which unfortunately failed to converge, likely due to the small sample size, resulting in invalid inferences (data not shown). While there is cumulative evidence to suggest that gut microbes could affect host metabolism (Jonsson & Backhed, 2017), its role in atherosclerosis progression is still unclear. Changes in the gut microbiome could promote atherosclerosis by several pathways, such as (a) activation of lipopolysaccharide (LPS)-mediated inflammation, (b) increased cholesterol or bile acid levels, or (c) increased gut-derived pro-atherogenic metabolites (Jonsson & Backhed, 2017). Thus, Kasahara et al reported that germ-free ApoE^−/−^ mice exhibited reduced atherosclerotic lesions in comparison to conventional ApoE^−/−^ mice, likely due to lower plasma LPS levels and decreased proinflammatory cytokine expression in the aorta (Kasahara et al., 2017). While we did not observe elevated plasma LPS (data not shown) or lipid levels (Table 1) in PM-exposed mice, our data suggests the possibility that other gut microbiota-derived metabolites could perhaps be responsible for the increased atherosclerotic lesions and/or triggering ER stress pathways, thereby overpowering the protective effects of SCFAs. Thus, it is known that some bacterial species promote atherosclerosis *via* production of trimethylamine N-oxide (TMAO) (Gregory et al., 2015; Wang et al., 2011) by flavin-containing monooxygenase3 (*Fmo3*), which oxidizes trimethylamine (TMA), derived from gut flora metabolism of choline to TMAO (Bennett et al., 2013). Unfortunately, we could not measure plasma TMAO levels due to limited sample availability, but we did observe an almost significant increase in the hepatic mRNA levels of *Fmo3* in PM-exposed mice (p = 0.06, Fig. S11), suggesting the possibility of increased enzymatic production of TMAO. Interestingly, TMAO has been shown to trigger ER stress by activating the protein kinase RNA-like ER kinase (PERK) signaling pathway of UPR, and interventions to reduce TMAO, either by manipulation of the gut microbiota or by inhibition of *Fmo3* can reduce PERK activation in the liver (Chen et al., 2019). Thus, future studies are warranted to assess the potential role of TMAO in PM-induced gut microbiota dysbiosis, increased ER stress, and worsened atherosclerosis. Interestingly, however, Stepankova et al have reported that germ-free ApoE^−/−^ mice fed a low cholesterol diet developed larger atherosclerotic lesions in comparison to conventional ApoE^−/−^ mice instead, suggesting that the presence of certain commensal gut bacteria could exhibit a protective role in atherosclerosis development (Stepankova et al., 2010). Therefore, it remains to be determined if changes in the gut microbiome contribute or protect against PM-induced atherosclerosis, which will likely depend on the type of alterations induced by PM.

## 5. Limitations

Our study has several limitations. Firstly, our assessment of the gut microbiome did not resolve the individual species within the genera, and it appears that several of these effects could be exhibited by various members of the same genera. Secondly, the associations between changes in the gut microbiome, worsened atherosclerosis and other parameters are only suggestive and do not prove causality, so further confirmation and mechanistic studies including fecal microbiota transplantation in antibiotic-depleted and gnotobiotic germ-free mice are warranted to establish causality. Thirdly, while we performed adjustments for cage effects and statistically significant taxa, the associations between microbiome abundance and other parameters were not adjusted for multiple comparisons. Lastly, our study only assessed male mice in the ApoE^−/−^ background; therefore, future studies should include females as well to determine the potential for sex in microbial effects.

## 6. Conclusions

Sub-chronic inhalation exposure to re-aerosolized PM, highly enriched in particles in the ultrafine size range exacerbated atherosclerotic lesion development in different segments of the aorta, together with marked alterations in the gut microbiota composition and fecal SCFAs, that correlated with worsened atherosclerosis. In addition, ultrafine PM also induced lipid peroxidation and ER stress in the liver, and a trend towards reduced plasma PON activity, which correlated with changes in the gut microbiome. Considering that associations between atherosclerotic lesions, hepatic ER stress and altered gut microbiota composition do not imply causality, future *in-vivo* studies using genetic knockout models or pathway inhibition experiments, together with testing cecal microbiota transplantation in germ-free ApoE^−/−^ mice are warranted to examine whether ultrafine PM-induced effects on atherosclerosis and hepatic ER stress are mediated by alterations in the gut microbiota composition.

## Supplementary Material

1

## Figures and Tables

**Table 1:** Ultrafine PM exposure and lipids. Plasma and hepatic lipids in ultrafine PM and FA exposed mice. Each value is shown as mean ± SEM. Statistical significance between the two groups were determined using unpaired Student’s *t*-test for plasma lipids and hepatic total cholesterol, and Mann Whitney’s *U* test for hepatic triglycerides.

Lipids	FA	PM	p-value
Plasma lipids, mg/dL^#^			
Total cholesterol	500.7 ± 23.9	503.1 ± 31.7	0.95
Triglycerides	53.2 ± 4.5	51.7 ± 4.5	0.80
HDL cholesterol	23.3 ± 1.2	20.3 ± 1.6	0.16
Unesterified cholesterol	203.3 ± 11.6	210.0 ± 17.2	0.75
Free fatty acids	24.0 ± 1.7	24.8 ± 2.1	0.77
Hepatic lipids, μg/mg protein^$^			
Total cholesterol	25.9 ± 0.6	27.0 ± 1.0	0.36
Triglycerides	170.8 ± 13.8	158.2 ± 5.7	0.86

# n=9(FA and PM)

$ n=10(FA), n=11(PM).

## Data Availability

Data will be made available on request.

## Appendix A. Supplementary material

Supplementary data to this article can be found online at https://doi.org/10.1016/j.envint.2025.109964.
