# Supplementary material for: Ultrafine particulate matter exposure induces gut microbiota dysbiosis together with ER stress in the liver and worsened atherosclerosis
Source: Environ Int. Author manuscript; Available in PMC 2026 Jul 15. (PMC13372509; doi:10.1016/j.envint.2025.109964)
Supplement: 1 [file NIHMS2187902-supplement-1.pdf]

Ultrafine Particulate Matter Exposure Induces Gut Microbiota Dysbiosis Together with ER Stress in the Liver and Worsened Atherosclerosis

Rajat Gupta [a, b, c, #], Candace Chang [a, b, c, d, #], David H. Gonzalez [a, c], Priyansha Srivastava [a], Collin Le [d], Daniel P. Stefanko [e], Jocelyn A. Castellanos [a, b], Mohamad Navab [a], Srinivasa T. Reddy [a, c, f, g], Gregory A. Fishbein [e], Constantinos Sioutas [h], Jonathan P. Jacobs [c, d, i, j], Tzung Hsiai [a, k], Jesus A. Araujo [a, b, c, g, *]

[a]Division of Cardiology, David Geffen School of Medicine, University of California Los Angeles, Los Angeles, California, USA.
[b]Department of Environmental Health Sciences, Fielding School of Public Health, University of California Los Angeles, Los Angeles, California, USA.
[c]Environmental and Molecular Toxicology Interdepartmental Program, University of California Los Angeles, Los Angeles, California, USA.
[d]Vatche and Tamar Manoukian Division of Digestive Diseases, David Geffen School of Medicine, University of California Los Angeles, Los Angeles, California, USA.
[e]Department of Pathology, David Geffen School of Medicine, University of California Los Angeles, Los Angeles, California, USA.
[f]Molecular & Medical Pharmacology, University of California Los Angeles, Los Angeles, California, USA.
[g]Molecular Biology Institute, University of California Los Angeles, Los Angeles, California, USA.
[h]University of Southern California, Viterbi School of Engineering, Los Angeles, California, USA.
[i]Goodman-Luskin Microbiome Center, University of California Los Angeles, Los Angeles, California, USA.
[j]Division of Gastroenterology, Hepatology and Parenteral Nutrition, Veterans Administration Greater Los Angeles Healthcare System, Los Angeles, California, USA.
[k]Henry Samueli School of Engineering, University of California Los Angeles, Los Angeles, California, USA.

Running Title: Particulate Matter and Dysbiosis

***Corresponding author**: Jesus A. Araujo, MD, PhD. Division of Cardiology, Department of Medicine, David Geffen School of Medicine, University of California-Los Angeles, 10833 Le Conte Avenue, CHS 43-264, Los Angeles, CA 90095. P.O. Box 951679. Phone number (310) 825-3222, Fax number (310) 206-9133. E-mail address: JAraujo@mednet.ucla.edu

[#]equal contribution

Table S1: Histological scoring system for inflammation in the lungs by H&E staining# as described previously (Bayes et al., 2016; Gori et al., 2019).

Score	Alveolar Involvement
0	None
1	Mild (patchy increased cellularity without septal thickening)
2	Moderate (increased cellularity with septal thickening)
3	Severe (25-50% visualized lung with increased cellularity/thickening)
4	Diffuse (>50% visualized lung with increased cellularity/thickening)

#n=10 (FA=5 and Ultrafine PM=5).

<u>Table S2</u>: qPCR Taqman gene expression assay IDs for mouse targets.

S. No.	Gene	Taqman Assay ID#
1.	*Il-1α*	Mm00439620_m1
2.	*Il-1β*	Mm00434228_m1
3.	*Il-6*	Mm00446190_m1
4.	*Tnfα*	Mm00443258_m1
5.	*Xbp1*	Mm00457357_m1
6.	*Atf6*	Mm01295319_m1
7.	*Chop*	Mm01135937_g1
8.	*Nqo1*	Mm01253561_m1
9.	*Catalase*	Mm00437992_m1
10.	*β-actin*	Mm02619580_g1

#Primer sequences are not available.

Table S3: Chemical characterization and profile of particulate matter (PM) aerosol.

	Concentration (ng/g)
**Trace Elements and Metals**	
Ca	4468.72
S	3120.13
Al	2826.18
Na	2316.78
Fe	2277.93
K	1817.04
Mg	865.37
Zn	413.22
Ba	318.48
Cu	186.41
Ti	142.35
P	139.35
Sr	68.45
Mn	59.63
Pb	54.31
Sb	33.77
Ni	16.38
B	15.11
Zr	14.90
Sn	13.66
Cr	12.16
V	7.62
Mo	7.26
Li	4.20
Rb	3.64
Ce	2.63
Co	1.68
As	1.62
La	1.35
Nd	0.90
Cd	0.88
Ag	0.84
W	0.68
Y	0.67
Hf	0.34
Nb	0.32

Th	0.27
Pr	0.23
Cs	0.15
Sm	0.14
Pd	0.13
Dy	0.10
U	0.10
Eu	0.07
Yb	0.06
Tl	0.05
Pt	0.03
Ho	0.02
Rh	0.02
Lu	0.01
**Inorganic Ions**	**% PM mass**
Cl	1.24
NO_3	16.20
PO_4	0.07
SO_4	11.34
Na	1.13
NH_4	2.58
K	0.55
**Total composition**	**% PM mass**
Metals and elements	19.22
Water-soluble inorganic ions	33.11
Total Carbon[#]	47.69

[#]includes sum of organic and elemental inorganic carbon.

Table S4: Tissue and BALF levels of HETEs and HODEs[$].

	FA	PM	p-value
**Plasma, ng/mL[#]**			
5-HETE	3.20 ± 0.25	3.30 ± 0.27	0.93
12-HETE[a]	37 ± 9.10	35.40 ± 12.80	0.53
15-HETE[a]	1.01 ± 0.16	1.14 ± 0.32	0.63
9-HODE	9.12 ± 0.74	9.93 ± 0.85	0.48
13-HODE[a]	15.50 ± 1.73	15.60 ± 1.83	0.91
**Liver, ng/mg protein[@]**			
5-HETE	0.14 ± 0.03	0.09 ± 0.01	0.17
12-HETE[a]	0.48 ± 0.30	0.46 ± 0.16	0.33
15-HETE[a]	0.31 ± 0.07	0.27 ± 0.03	>0.99
9-HODE	4.80 ± 0.64	3.90 ± 0.23	0.16
13-HODE	3.80 ± 0.40	3.50 ± 0.30	0.59
**BALF, ng/mg protein†**			
5-HETE	0.13 ± 0.02	0.14 ± 0.03	0.69
12-HETE[a]	5.28 ± 2.08	6.72 ± 1.84	0.78
15-HETE[a]	0.30 ± 0.09	0.46 ± 0.13	0.20
9-HODE[a]	0.99 ± 0.19	1.16 ± 0.21	0.60
13-HODE[a]	1.40 ± 0.29	1.31 ± 0.18	0.90

FA indicates filtered air; PM, ultrafine particulate matter; HETE, hydroxyeicosatetraenoic acid; HODE, hydroxyoctadecadienoic acid; and BALF, bronchoalveolar lavage fluid.

Statistical significances between the FA and PM groups were determined using unpaired Student's t-test, except for the biomarkers with "[a]", which were analyzed using Mann Whitney's *U* test, with *p* values as indicated above.

[$]Results are expressed as mean ± SEM.

[#]n=10 for each group

[@]n=9 for FA and n=11 for PM

†n=8 for FA and n=11 for PM

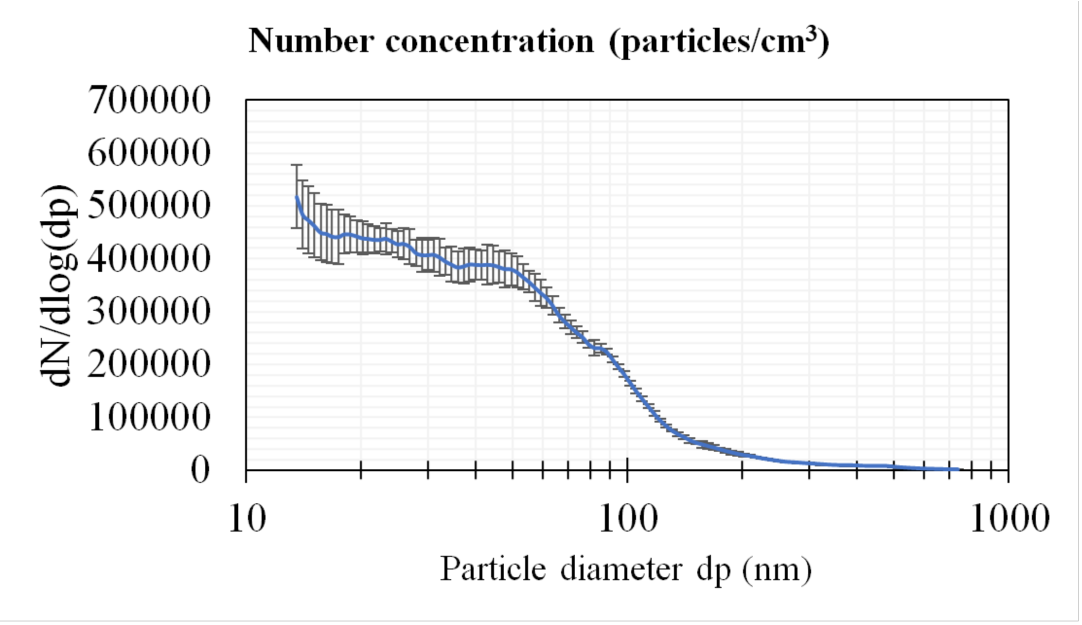

<u>Figure S1</u>. Average particle size distributions in the re-aerosolized PM aerosol.

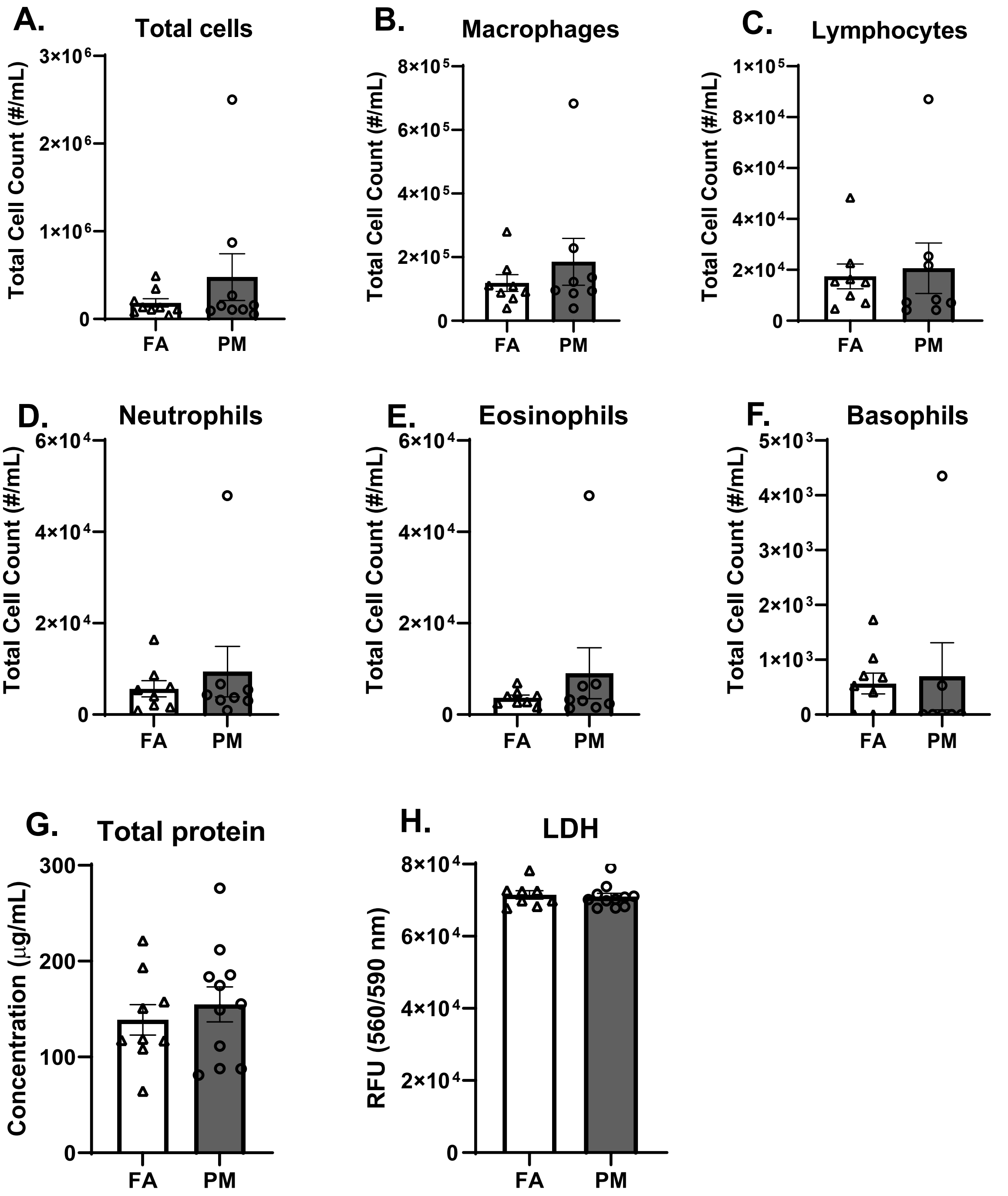

<u>Figure S2</u>. Characterization of bronchoalveolar lavage fluid. Cell differentials were determined in the bronchoalveolar lavage fluid (BALF) and normalized by volume in ultrafine PM and FA-exposed mice for **(A)** Total cells, **(B)** Macrophages, **(C)** Lymphocytes, **(D)** Neutrophils, **(E)** Eosinophils, and **(F)** Basophils. **(G)** Total protein and **(H)** Lactate dehydrogenase (LDH) levels were also obtained in the BALF. Assessment was performed in samples from individual mice, n=8-11/group. Each bar denotes mean ± SEM. Statistical significance between the two treatment groups were determined using Student's t test.

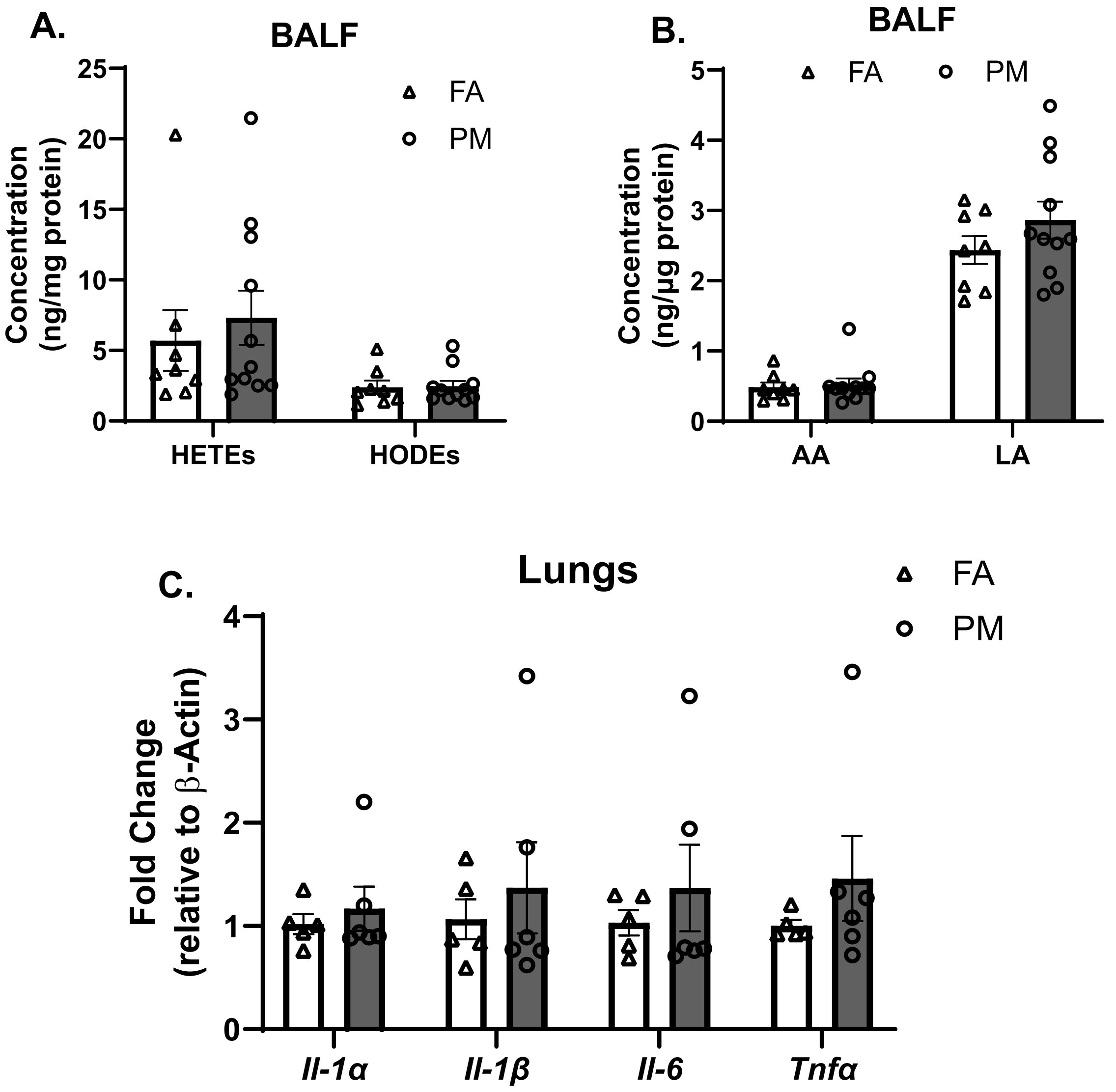

<u>Figure S3</u>. **Ultrafine PM exposure and oxidized fatty acid metabolites in the BALF, and mRNA levels of proinflammatory cytokines in the lungs. (A)** Concentrations of HETEs (Sum total of 5-, 12- and 15-HETEs) and HODEs (Sum total of 9- and 13-HODEs), and **(B)** their parental polyunsaturated fatty acids including arachidonic acid (AA) and Linoleic Acid (LA), respectively in the Bronchoalveolar Lavage Fluid (BALF). **(C)** mRNA levels expressed as fold change of proinflammatory cytokines as measured by qPCR in the lungs of ultrafine PM *vs.* FA-exposed mice. Each bar denotes mean ± SEM, n=8 (FA) and n=11 (PM) for Panels **(A)** and **(B)**, and n=5 (FA) and n=6 (PM) for Panel **(C)**. Statistical significance between the two treatment groups were determined using Student's t test. HETEs, Hydroxyeicosatetraenoic acids; HODEs, Hydroxyoctadecadienoic acids.

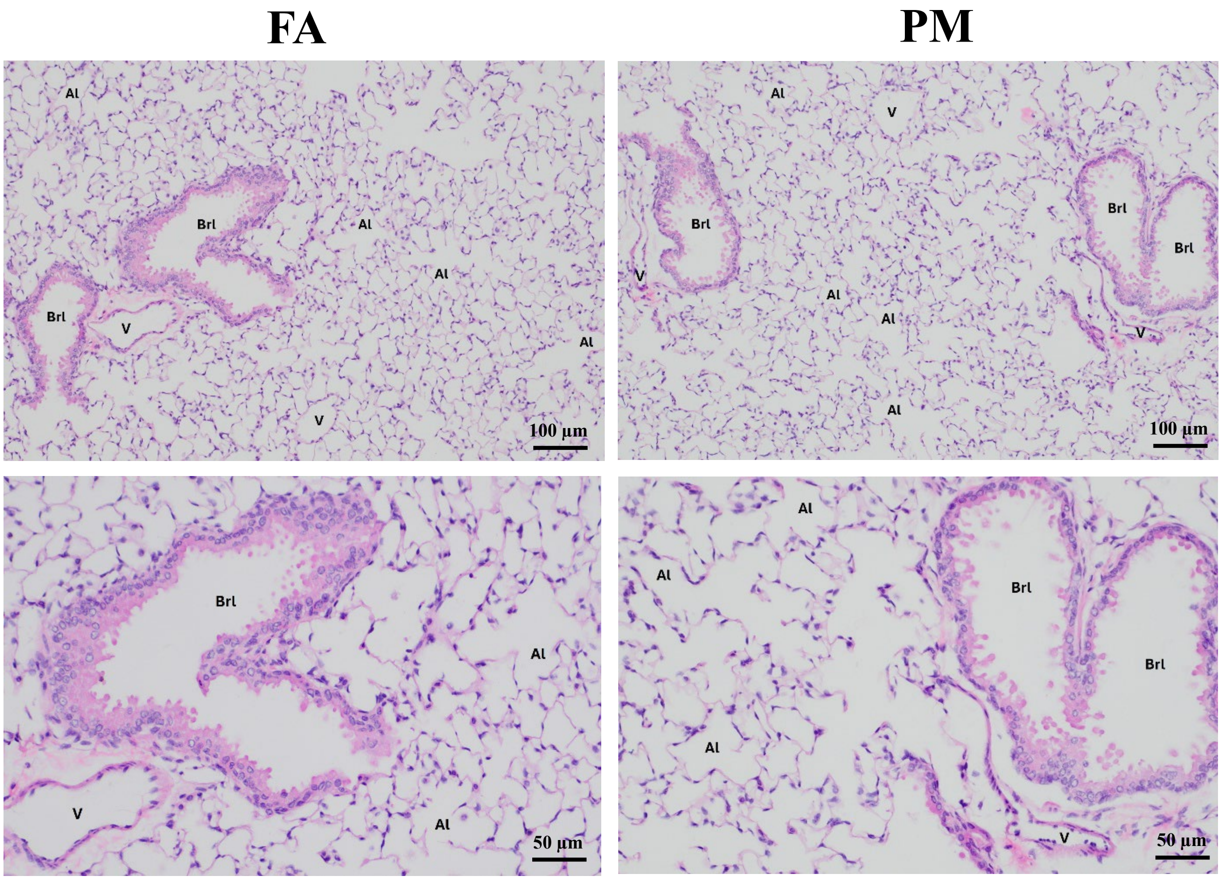

<u>Figure S4</u>. Lung Histology. Histological analysis of representative lung sections by staining with hematoxylin and Eosin (H&E) showing lung cellular structure from mice exposed to filtered air (FA) or ultrafine particulate matter (PM). Upper panel shows images at 100x magnification and lower panel shows images at 200x magnification. n=5 samples from each group were randomly selected for histological assessment. Scale bars indicate 100 µm for 100x magnification and 50 µm for 200x magnification. Al: Alveolus, V: Blood vessel, Brl: Bronchiole.

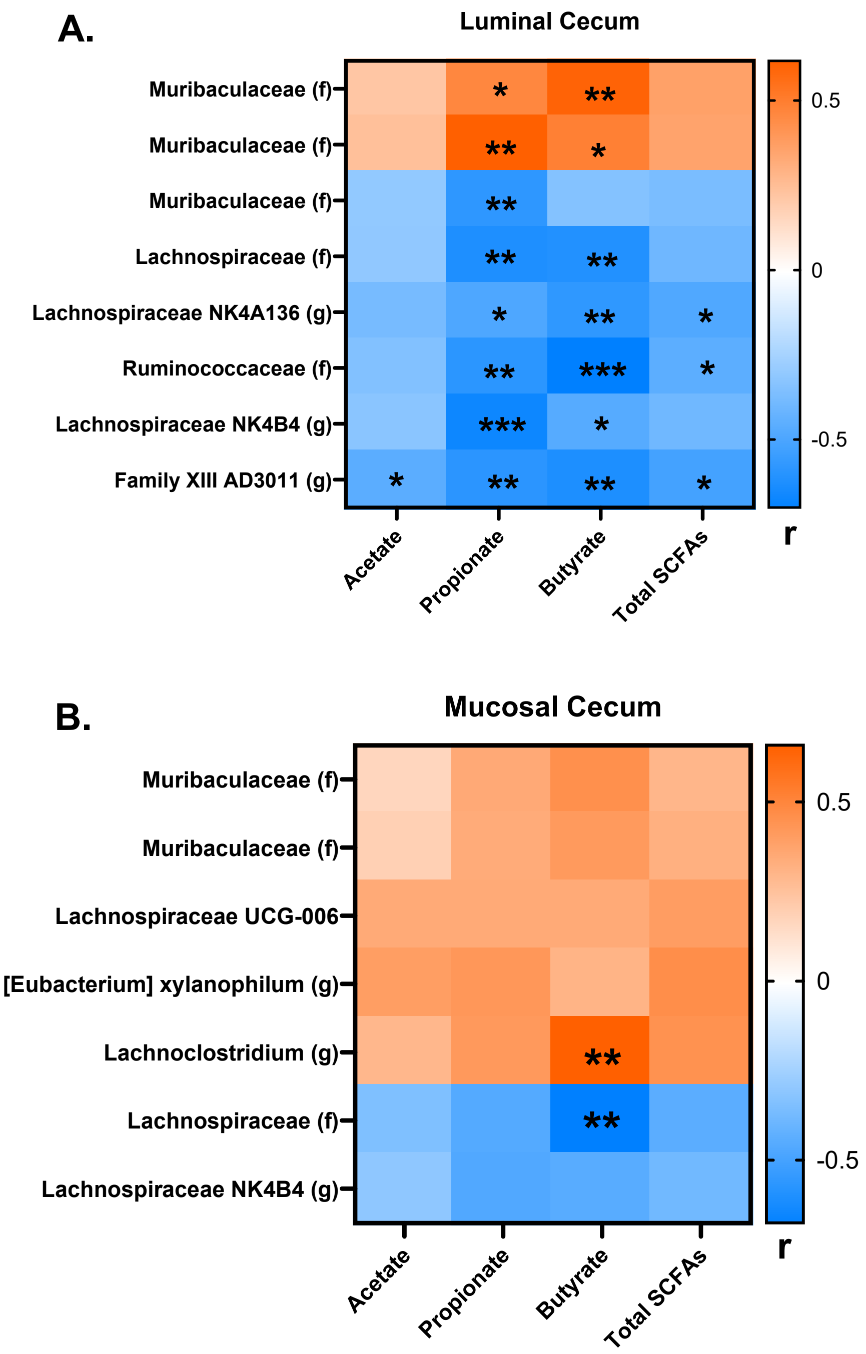

<u>Figure S5</u>. Associations between cecal microbiota abundance and fecal short chain fatty acids. Heat maps representing Spearman's correlation analysis between the relative abundance of **(A)** luminal and **(B)** mucosal cecal microbiome with fecal short chain fatty acids (SCFAs) in the ultrafine PM and FA-exposed mice. Orange colors indicate positive associations, whereas blue colors indicate negative associations based on the correlation coefficient (r) as shown. All the microbial taxa shown in the heat maps for luminal and mucosal cecal microbiome correspond to the differentially abundant taxa. Significant associations are denoted by asterisks. *p<0.05, **p<0.01 and ***p<0.001, n=10-11/group.

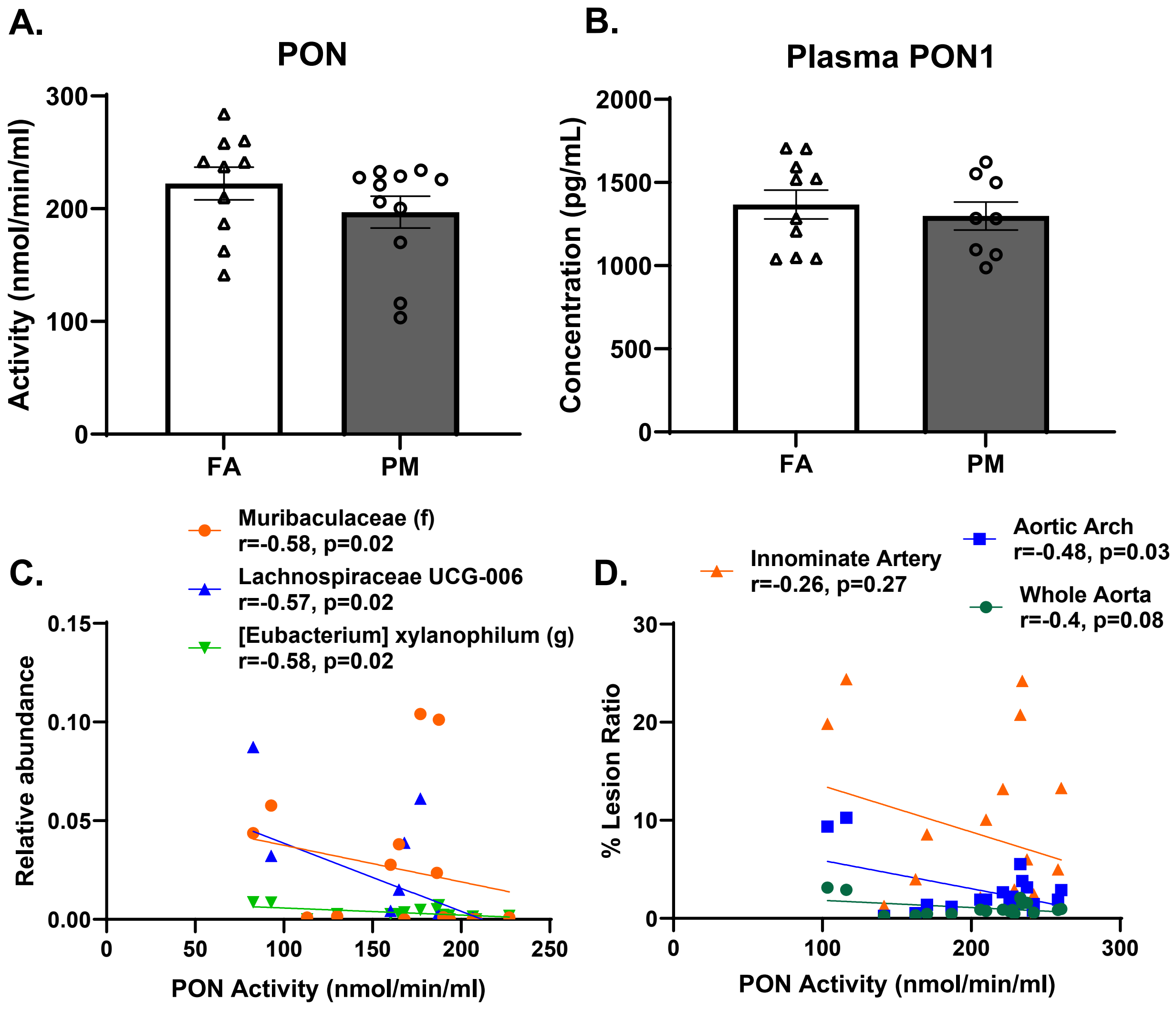

<u>Figure S6</u>. **Plasma paraoxonase activity, mass concentration and associations with atherosclerotic lesions and mucosal cecal microbiota abundance. (A)** Plasma paraoxonase (PON) functionality was determined by assessing PON activity in ultrafine PM and FA exposed mice. **(B)** PON1 mass concentration. Each bar denotes mean ± SEM (n=10 (FA) and n=11 (PM) for Panel **(A)** and n=10 (FA) and n=8 (PM) for Panel **(B)**). Statistical significance between the two groups were determined using Mann-Whitney's U test for Panel **(A)** and Student's t-test for Panel **(B)**. **(C)** Spearman's correlation analysis indicating significant negative associations between PON activity and differentially abundant mucosal cecal microbiota. **(D)** Pearson's correlation analysis between PON activity and %lesion ratio in the innominate artery, aortic arch and whole aorta of ultrafine PM and FA-exposed mice. *p* values and correlation coefficients (r) are shown above.

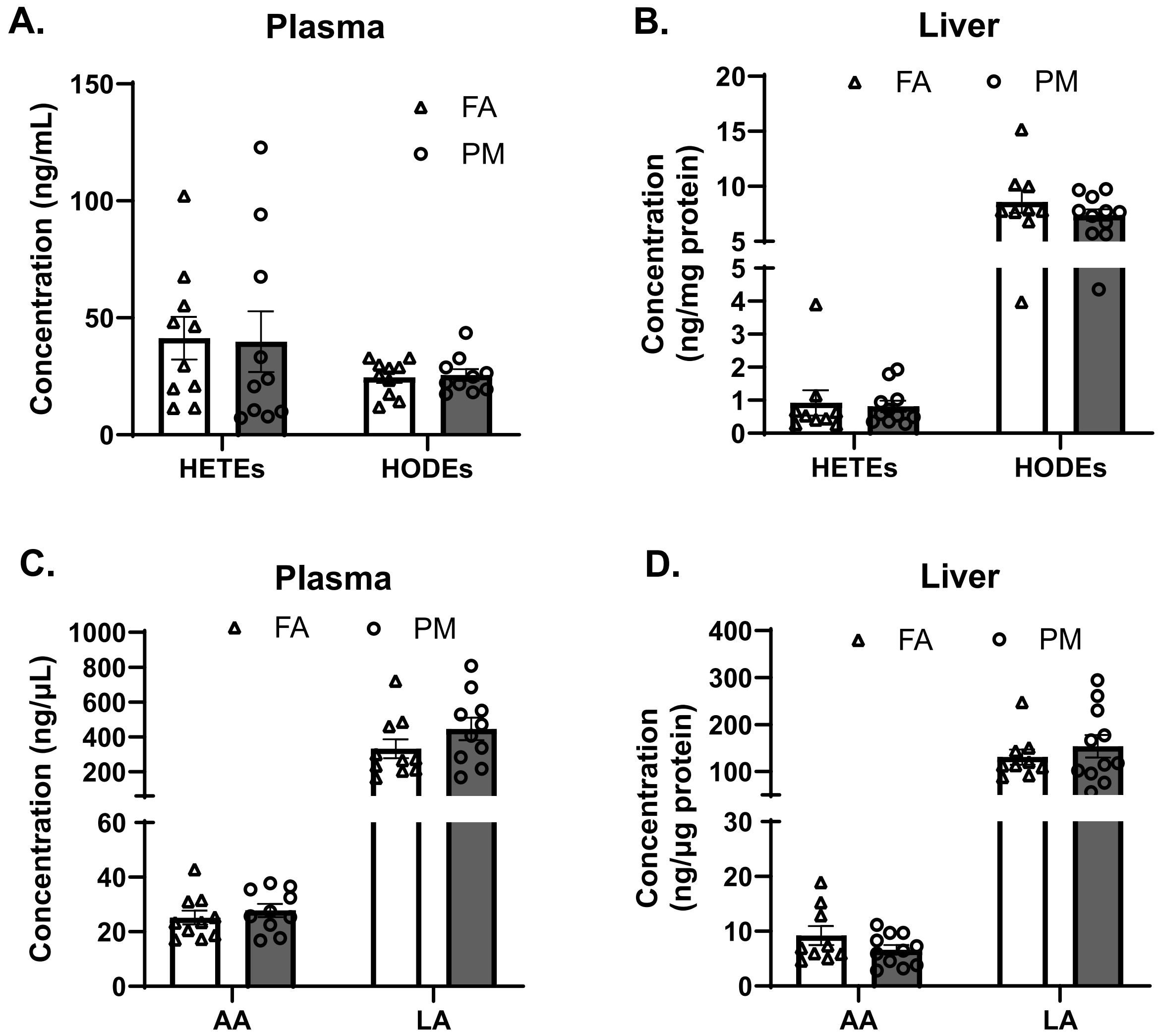

Figure S7. Ultrafine PM exposure and oxidized fatty acids metabolites in plasma and liver. Concentrations of HETEs (Sum total of 5-, 12- and 15-HETEs) and HODEs (Sum total of 9- and 13-HODEs) in the **(A)** plasma and **(B)** liver, and their parental polyunsaturated fatty acids including arachidonic acid (AA) and Linoleic Acid (LA), respectively in the **(C)** plasma and **(D)** liver of ultrafine PM and FA-exposed mice. Each bar denotes mean ± SEM (n=10 (FA) and n=10 (PM) for Panels **(A)** and **(C)**, and n=9 (FA) and n=11 (PM) for Panels **(B)** and **(D)**). Statistical significance between the two treatment groups were determined using Student's t test. HETEs, Hydroxyeicosatetraenoic acids; HODEs, Hydroxyoctadecadienoic acids.

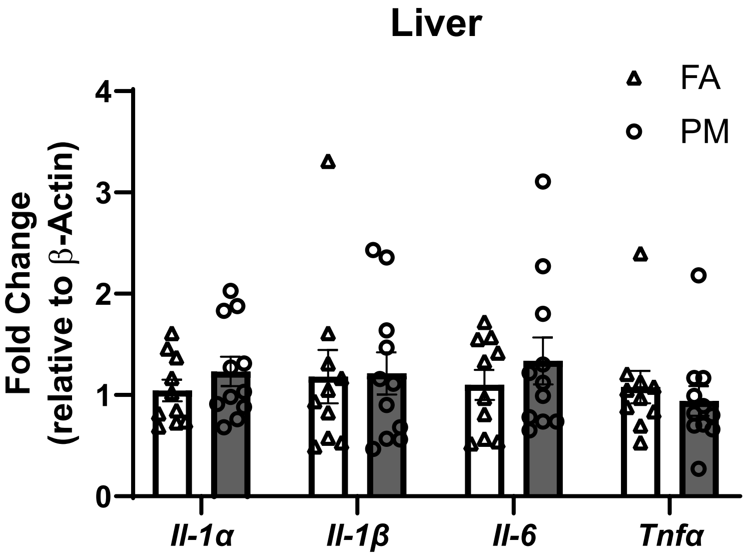

<u>Figure S8</u>. Ultrafine PM exposure and proinflammatory cytokine gene expression. mRNA levels expressed as fold change of proinflammatory cytokines measured by qPCR in the liver of ultrafine PM *vs.* FA-exposed mice. Each bar denotes mean ± SEM (n=10 (FA) and n=11 (PM)). Statistical significance between the two treatment groups was determined using Student's t test.

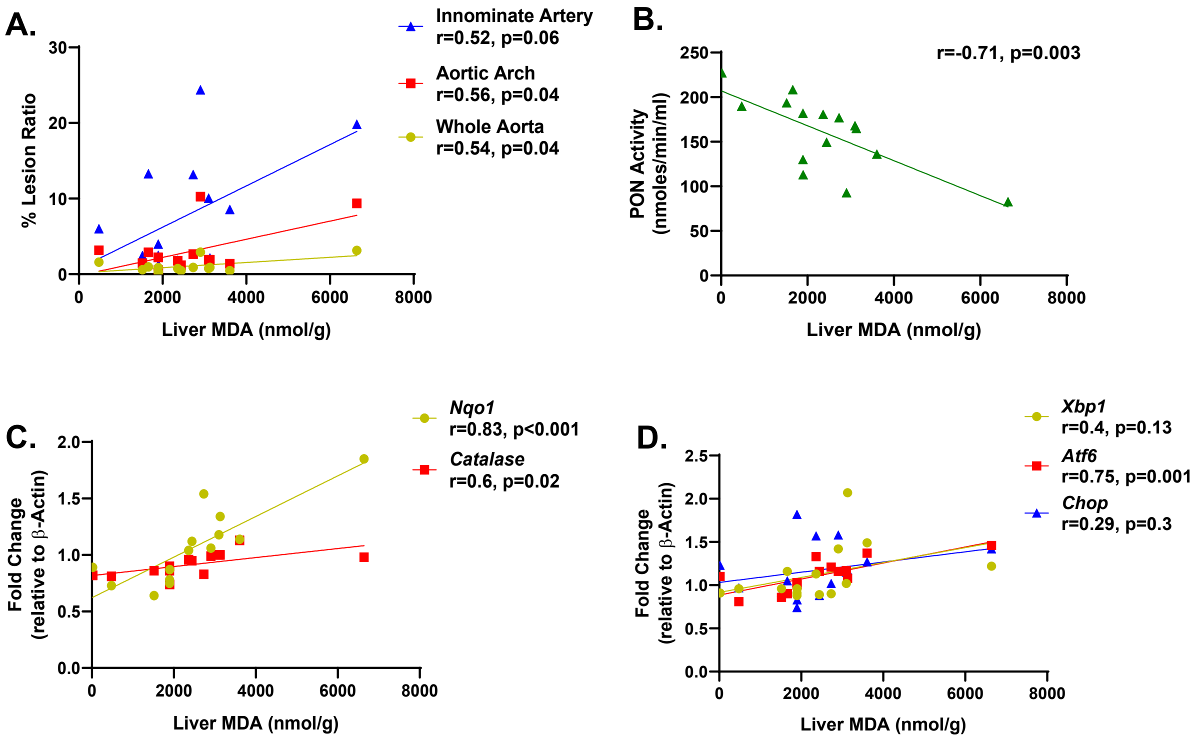

<u>Figure S9</u>. Associations between liver MDA and systemic vascular effects. Pearson's correlation analysis between liver MDA levels and **(A)** %lesion ratio in the innominate artery, aortic arch and whole aorta, **(B)** PON activity in the plasma, hepatic mRNA levels expressed as fold change of **(C)** antioxidant genes including *Nqo1* and *Catalase*, and **(D)** ER stress marker genes including *Xbp1*, *Atf6* and *Chop* in ultrafine PM and FA-exposed mice (n=8 (FA) and n=7 (PM) for Panels **(A)** and **(C)**, and n=9 (FA) and n=7 (PM) for Panels **(B)** and **(D)**). *p* values and correlation coefficients (r) are as shown.

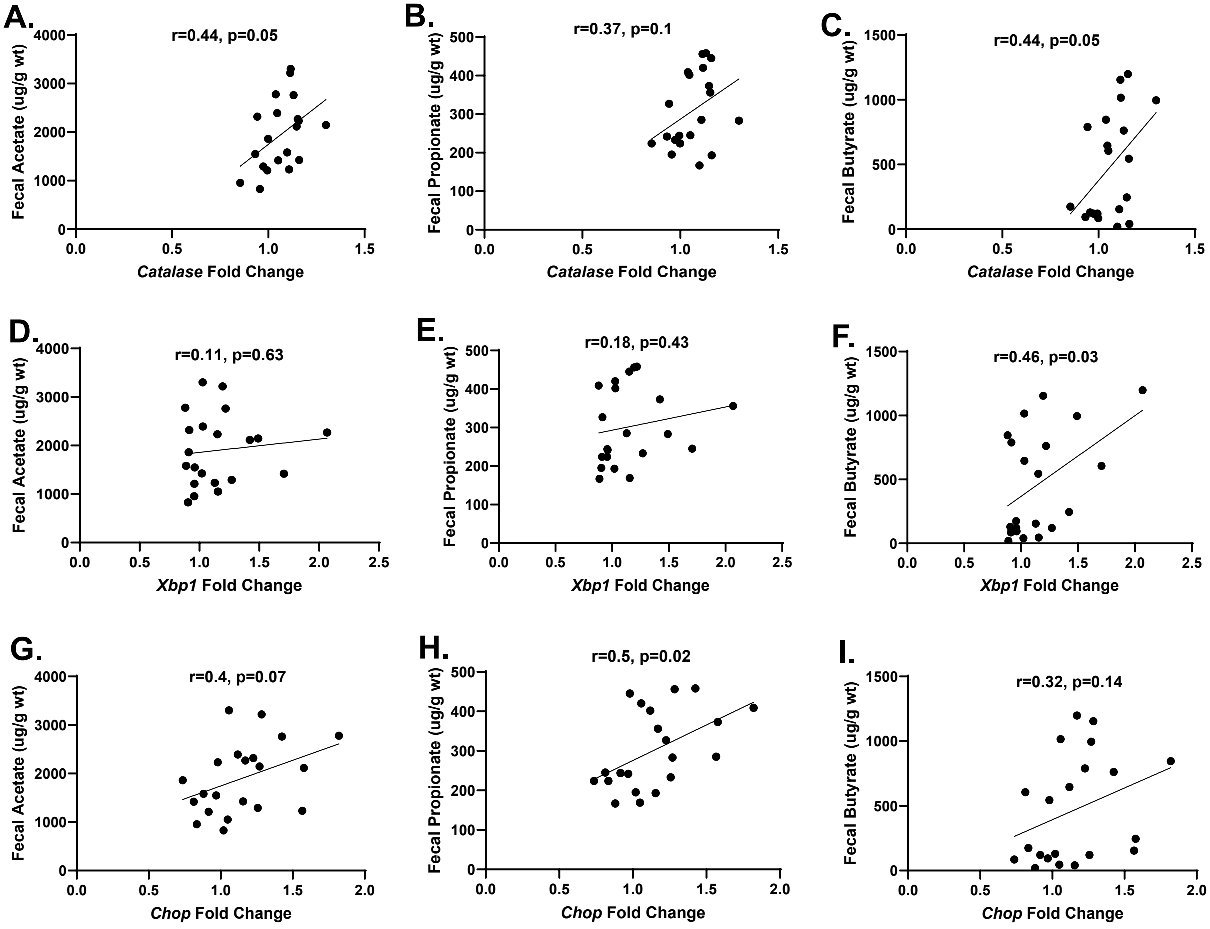

Figure S10. **Associations between fecal SCFAs, ER stress and antioxidant gene expression.** Pearson's correlation analysis between mRNA levels of *Catalase* expressed as fold change with fecal SCFA levels including **(A)** Acetate, **(B)** Propionate and **(C)** Butyrate, mRNA levels of *Xbp1* with fecal **(D)** Acetate, **(E)** Propionate and **(F)** Butyrate, and mRNA levels of *Chop* with fecal **(G)** Acetate, **(H)** Propionate and **(I)** Butyrate in ultrafine PM and FA-exposed mice. *p* values and correlation coefficients (r) are as shown.

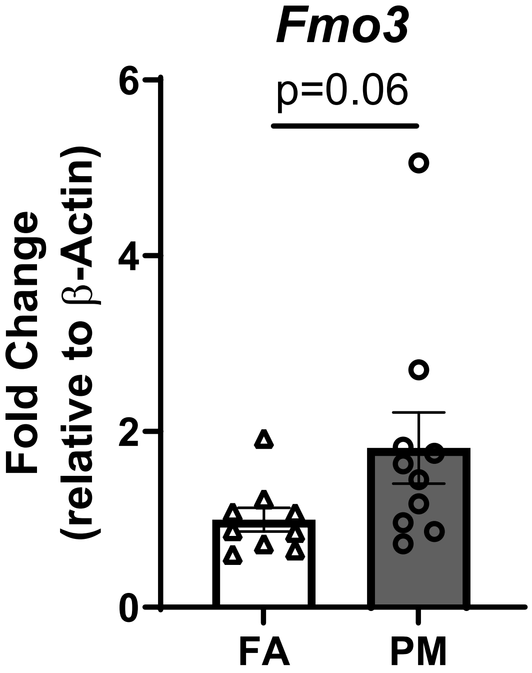

Figure S11. Ultrafine PM exposure and *Fmo3* gene expression. mRNA levels expressed as fold change of flavin-containing monooxygenase3 (*Fmo3*) measured by qPCR in the liver of ultrafine PM *vs.* FA-exposed mice. Each bar denotes mean ± SEM (n=9 (FA) and n=10 (PM)). Statistical significance between the two treatment groups were determined using Mann Whitney's U test.

References

Bayes, H. K., et al. (2016). A murine model of early Pseudomonas aeruginosa lung disease with transition to chronic infection. *Scientific Reports, 6*(1), 35838. doi:10.1038/srep35838

Gori, S., et al. (2019). Acetylcholine-treated murine dendritic cells promote inflammatory lung injury. *PLoS One, 14*(3), e0212911. doi:10.1371/journal.pone.0212911
